# Parahydrogen-induced polarization allows 2000-fold signal enhancement in biologically active derivatives of the peptide-based drug octreotide

**DOI:** 10.1038/s41598-023-33577-2

**Published:** 2023-04-19

**Authors:** Jonas Lins, Yuliya A. Miloslavina, Stefania C. Carrara, Lorenz Rösler, Sarah Hofmann, Kevin Herr, Franziska Theiß, Laura Wienands, Olga Avrutina, Harald Kolmar, Gerd Buntkowsky

**Affiliations:** 1 Eduard-Zintl-Institute for Inorganic and Physical Chemistry, Technische Universität Darmstadt, Alarich-Weiss-Straße 8, 64287 Darmstadt, Germany; 2 Clemens-Schöpf-Institute for Organic Chemistry and Biochemistry, Technische Universität Darmstadt, Alarich-Weiss-Straße 4, 64287 Darmstadt, Germany

**Keywords:** Solution-state NMR, Cancer imaging

## Abstract

Octreotide, a somatostatin analogue, has shown its efficacy for the diagnostics and treatment of various types of cancer, i.e., in octreotide scan, as radio-marker after labelling with a radiopharmaceutical. To avoid toxicity of radio-labeling, octreotide-based assays can be implemented into magnetic resonance techniques, such as MRI and NMR. Here we used a Parahydrogen-Induced Polarization (PHIP) approach as a cheap, fast and straightforward method. Introduction of l-propargyl tyrosine as a PHIP marker at different positions of octreotide by manual Solid-Phase Peptide Synthesis (SPPS) led to up to 2000-fold proton signal enhancement (SE). Cell binding studies confirmed that all octreotide variants retained strong binding affinity to the surface of human-derived cancer cells expressing somatostatin receptor 2. The hydrogenation reactions were successfully performed in methanol and under physiologically compatible mixtures of water with methanol or ethanol. The presented results open up new application areas of biochemical and pharmacological studies with octreotide.

## Introduction

Nuclear magnetic resonance (NMR) spectroscopy and magnetic resonance imaging (MRI) are widely used in various scientific and technical applications, providing detailed information for structural determination^1,2^, reaction monitoring^3^, molecular dynamics^4^ and interactions^5^, or clinical diagnostics^6^. These techniques apply a magnetic field to an atomic nucleus (most commonly spin 1/2 nuclei, e.g., ^1^H, ^13^C, ^15^N) and radio frequency pulses to characterize the resonance frequency of the nuclear spin according to its chemical or environmental surroundings.

One of the biggest challenges in the application of magnetic resonance techniques, like NMR spectroscopy and MRI, is their low sensitivity, which results from the small relative population differences of the nuclear spin states on the order of about ${P}_{eq}\approx {10}^{-5}$ at ambient temperature and typical magnetic field strengths of a few Tesla. This means that only a very small fraction of the molecules in a sample contributes to the MR signal and the vast majority of the molecules are MR silent, because they do not generate a net signal. This constrains both techniques to either large sample concentrations or long measurement times. To overcome these limitations at thermal equilibrium, different hyperpolarization techniques have been developed, such as Dynamic Nuclear Polarization (DNP)^7^, Chemically Induced Dynamic Nuclear Polarization (CIDNP)^8^ as well as more specific Parahydrogen Induced Polarization (PHIP)^9–12^ and Signal Amplification by Reversible Exchange (SABRE)^13,14^. These techniques transfer the spin polarization from a system with higher polarization (e.g., radicals for (CI)DNP, parahydrogen for PHIP/SABRE) to the spin system of interest. In particular, there has been a growing interest in the hyperpolarization of biologically active compounds, with much attention being paid to parahydrogen-based methods in recent years^15–21^. The big advantage of PHIP is the rather low requirement on equipment and the fast and easy operation, compared with, e.g., DNP. This makes PHIP very cost- and time-effective. It relies on the transfer of polarization from para-enriched hydrogen gas onto the molecule of interest by a catalyzed hydrogenation with unsaturated carbon–carbon bonds, breaking the symmetry of the parahydrogen molecule, which would not be visible in the NMR otherwise. This way much of the initial spin order of parahydrogen is transformed into the very large nonequilibrium spin magnetization and transferred to the reaction product^9^. The reaction is magnetic field dependent and can be conducted at lower magnetic fields, typically the geomagnetic one, called ALTADENA^22^, or under high-field conditions directly inside the NMR magnet, designated as PASADENA. It has already been successfully implemented to certain biologically active substances^21,23^.

Most applications in the field of biologically active compounds focus on small molecules, such as pyruvate, which is involved in the energy metabolism, or fumarate^24,25^, which participates in the citric acid cycle and, as a hyperpolarized substrate, is of high diagnostic value for the control of tumor therapy^26^. The hyperpolarized compounds are generated from unsaturated precursors upon their reaction with parahydrogen and can be used as contrast agents in MRI for metabolic imaging^17,24^. Furthermore, they can help to study biological reactions and can be applied in drug development. Additionally, amino acids as precursors for neuro transmitters and as protein building blocks are suitable targets for PHIP^27,28^.

Peptides and proteins are able to bind specifically to certain targets. Hence, when combined with hyperpolarization techniques and MRI, they have a high potential for specific imaging applications and detailed protein studies down to physiological concentrations in NMR spectroscopy, such as, e.g., investigation of protein interactions. Since this class of biomolecules naturally does not possess any unsaturated carbon–carbon bonds needed for the reaction with parahydrogen, special building blocks with unsaturated moieties have to be introduced^16,18,21,29,30^.

**Figure 1:**
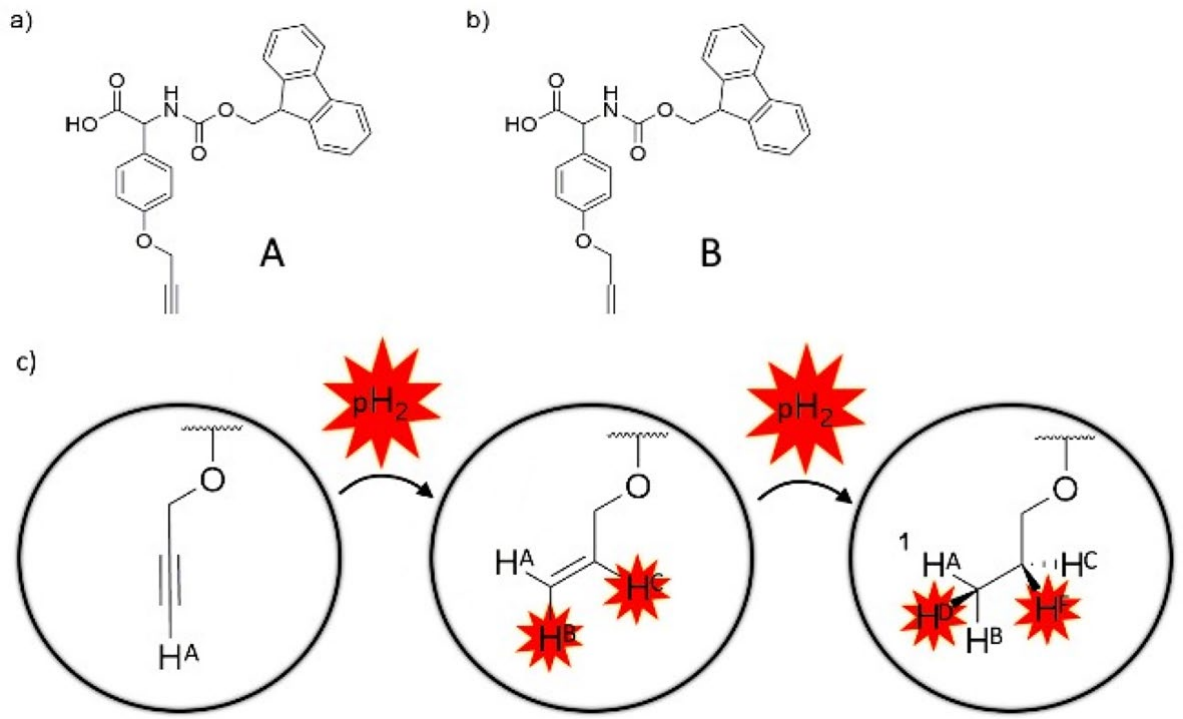
Structures of the incorporated unnatural amino acids used as PHIP markers. (a) Fmoc-l-propargyl tyrosine (**A**) and (b) Fmoc-l-allyl tyrosine (**B**). (c) The propargyl moiety (**A**) is hydrogenated to an allyl moiety (**B**) during the PHIP experiment and then further to a propyl moiety. The cis-orientation of hydrogens in **B** is supported by the action of a catalyst.

Our group has previously reported that PHIP can be applied to peptidic targets while preserving their biological activity, e.g., as antiplatelet aggregation inhibitor^31^ or as enzyme inhibitors^18,28^ Fmoc-l-propargyl tyrosine (**A**, Fig. 1) has been used as a building block and demonstrated excellent signal enhancement (SE) with a factor of 70, when incorporated into the cyclic oligopeptide SFTI-1 with 50% para-enriched hydrogen in ALTADENA-type experiments. With 90% para-enriched hydrogen in an automated PASADENA setup^32^ a strong increase in signal enhancement, up to 1200, has been observed, which provided enough sensitivity for single-shot ultrafast 2D-NMR of micromolar solutions^33^.

**Figure 2:**
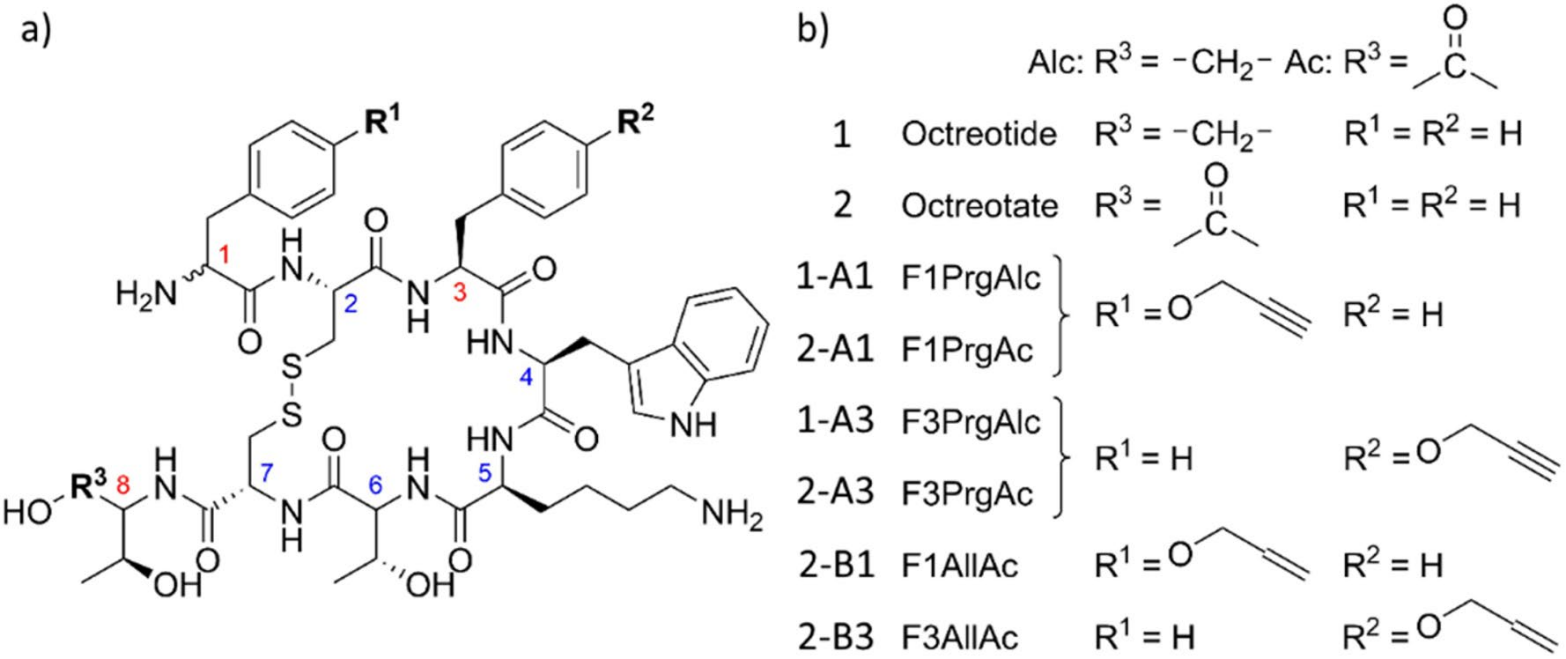
(**a**) General structures of octreotide (**1**) and octreotate (**2**) and its derivatives with a propargyl tyrosine (**A**) or allyl tyrosine (**B**) PHIP markers at position 1 (-**A1, B1**) or position 3 (-**A3, B3**). Compounds **2-B1** and **2-B3** represent the hydrogenation products of **2-A1** and **2-A3**. (**b**) Structure of labels and corresponding moieties.

In the present study, we demonstrate how hyperpolarization via PHIP can enhance the NMR signal of the octapeptide octreotide (**1**, Fig. 2) by three orders of magnitude while maintaining its bioactivity. Octreotide is a potent mimetic of the growth hormone inhibitor somatostatin^34,35^ and an important marker in Positron Emission Tomography (PET) and Computer Tomography (CT) (PET/CT) diagnostics^36^. In conjunction with a chelating ligand and a beta-decaying radio nuclide, octreotide conjugates have become a helpful tool for in vivo imaging and diagnostics of tumors that expresses certain somatostatin receptors (SSTR2, SSTR3 and SSTR5)^37–42^. Due to its smaller size and head-to-tail cyclization, it is much more stable than its natural paragon. As octreotide binds to the outer cell wall, no transport into the cell is necessary^43^.

These favorable PET/CT properties have led to astonishing improvements in the diagnostics and treatment of malignant tumors^44^, this makes octreotide an interesting target for magnetic resonance-based diagnostic techniques in biochemical and pharmacological research. As magnetic resonance techniques do not depend on the application of radioactive tracers and high doses of ionizing radiation that is harmful^45^, they are applicable in normal chemical laboratories without the need for radioisotopes, and any ionizing radiation to receive spectra of the chemical environment and high resolution images^6^. As the hyperpolarization by PHIP is time-limited to experiments on the order of seconds due to relaxation, possible application areas are, e.g., studies of fate and distribution of bioactive compounds as well as their metabolites in cell cultures or binding kinetics between octreotide and proteins. In certain cases, such as meningiomas, octreotide PET has been replaced by special MRI techniques, utilizing untargeted gadolinium-based contrast agents^46^. Octreotide in nanoparticles with gadolinium complexes has been successfully investigated as a possible targeted contrast agent for MRI^47,48^.

We synthesized and compared different modifications of octreotide, varying in the position of the PHIP marker and in the group on the *C*-terminus. PHIP experiments were done using an automated setup for PASADENA type experiments^32^. Parahydrogen was enriched to > 95% using a closed cycle helium refrigerator at 30 K and 10 bar while the reactions were conducted at a pressure of 7 bar. This should provide a theoretical 2.9-fold increase over the old setup utilizing 50% enriched parahydrogen at 77 K^49^. We achieved signal enhancement factors of up to 2000, which is, to the best of our knowledge, the highest enhancement for protons in peptides reported so far.

The modified compounds and the products of their hydrogenation were examined in a binding assay on lung cancer cells A549 and human embryonic kidney cells HEK293, both expressing the somatostatin receptors SSTR2 needed for binding octreotide and derivatives. We compared the binding affinity to the cells surface to unmodified octreotide and confirmed the retained bioactivity of all investigated compounds.

## Results and discussion

### Synthesis of octreotide and derivatives for PHIP applications

All variants, including the original octreotide (**1**, Fig. 2), were synthesized manually by solid-phase peptide synthesis (SPPS) on a 2-chlorotritylchloride resin using the Fmoc strategy^50^. After chain assembly, peptides were cleaved with TFA and cyclized by disulfide formation using hydrogen peroxide^51^ (see supporting information for detailed synthesis).

The structure of **1** has two possible sites where the PHIP marker **A** can be introduced, while preserving the original conformation of the molecule and its biological activity as close as possible. The marker can either replace the d-phenylalanine at position 1 or the l-phenylalanine at position 3, leading to the PHIP-labeled compounds OctF1PrgAlc (**1-A1**) or OctF3PrgAlc (**1-A3**), respectively. The -Alc in the name of these variants indicates the alcoholic *C* terminus, resembling a peptidol, as in the original structure of octreotide.

Certain somatostatin receptors have been found to possess a higher affinity to octreotate (**2**) derivatives bearing a carboxyl at the *C* terminus instead of an alcohol group^39^. Therefore, we synthesized two equally labeled compounds of octreotates resulting in the variants OctF1PrgAc (**2-A1**) and OctF3PrgAc (**2-A3**), where -Ac indicates the carboxylic acid at the C-terminus. To better investigate the hydrogenation products of **2-A1** and **2-A3**, l-allyl tyrosine (**B**) was introduced by SPPS to obtain the variants OctF1AllAc (**2-B1**) and OctF3AllAc (**2-B3**). As these also represent the active hyperpolarized form of the investigated octreotide derivatives, they were particularly interesting for the binding studies on human cell lines.

### Hydrogenation and observation of PHIP signal enhancement

In order to induce the PHIP effect, all compounds with propargyl tyrosine, **1/2-A1** and **1/2-A3** were hydrogenated, yielding the corresponding allyl variants, in which the triple bond, introduced with **A**, was reduced to a double bond.

**Figure 3:**
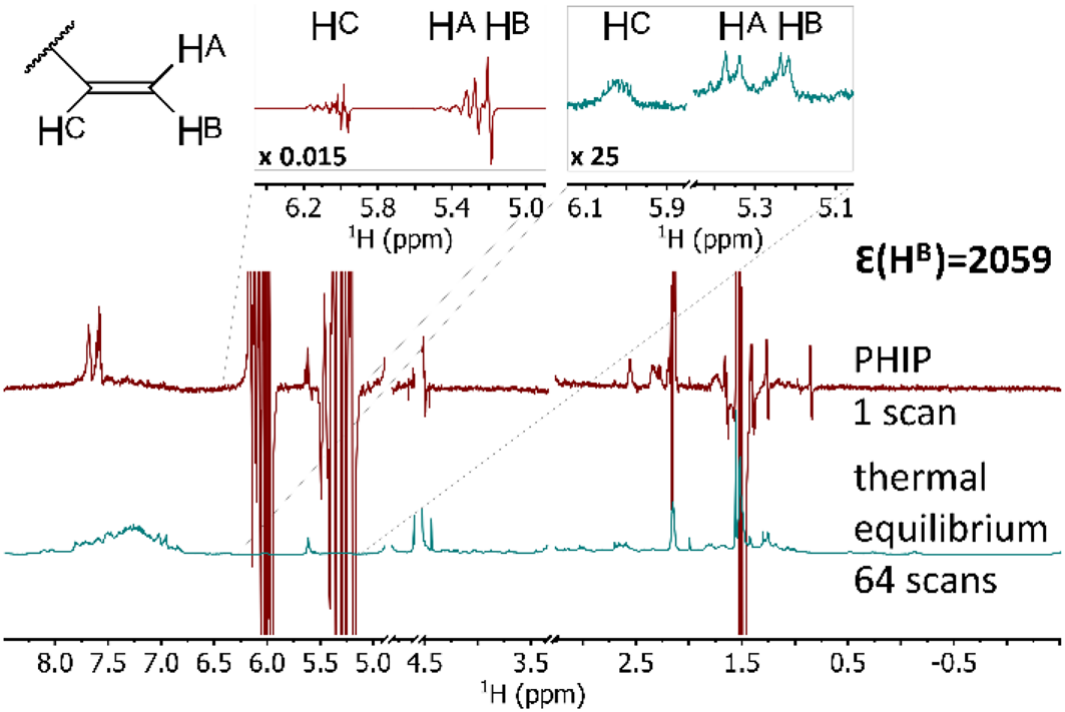
^1^H-NMR spectra of 1 mM hydrogenated OctF1PrgAlc (**1-A1**) in MeOD-d_4_ with 1.96 mM catalyst at 25 °C and 500 MHz (11.7 T). The upper spectrum in red is the PHIP spectrum after 25 s of reaction time, acquired in a single scan with a receiver gain of 3.9. The lower one in blue is the spectrum at thermal equilibrium after the relaxation of the hyperpolarization, acquired with 64 scans and a receiver gain of 287. The inserts (on the top) show a magnification of the signals of the allyl moiety. The hyperpolarized signals below 2.5 ppm correspond to the activated catalyst. The structure of the allyl moiety, which is a part of the reaction product of the PHIP marker, is presented on the top left. Hydrogens H^B^ and H^C^ originate from the parahydrogen molecule. The signals of H^A^ and H^B^ can be easily distinguished by their dipolar coupling constants to H^C^^53^. The greatest SE factor Ɛ of 2059 is obtained for the signal of hydrogen H^B^. The signals of the solvent, molecular hydrogen and a catalyst signal in the insert of the thermal spectrum have been removed for clarity of presentation.

For the reaction products, three hyperpolarized signals around 5.23 ppm, 5.35 ppm and 6.02 ppm were observed (Fig. 3). The signals at 5.23 ppm and 6.02 ppm stem from the hydrogen atoms H^B^ and H^C^, respectively (Fig. 1), which were added in the reaction. H^B^ generally showed the highest absolute integrals in the PHIP spectra and thus the highest signal enhancements. The T_1_ time of H^B^ as determined by inversion recovery experiments at 11.7 T in MeOD-d_4_ was about 3.60 (seconds) s for **2-B1** and 3.14 s for **2-B3**. H^C^ showed somewhat smaller absolute integrals due to its stronger coupling with neighboring protons in the molecule. This leads to broadening of the signal and partial canceling of some parts with opposing phase. Its T_1_ times were 3.59 s for **2-B1** and 2.55 s for **2-B3**. The H^A^ proton at 5.35 ppm is hyperpolarized due to polarization transfer from H^B^ and H^C^ via coupling and cross relaxation effects^52^. Its hyperpolarization emerged slowly after the end of the reaction and was generally weaker. T_1_ was 2.99 s for **2-B1** and 4.80 s for **2-B3**. The here reported signal enhancements focus on those acquired for H^B^ (Table 1). The enhancement factors of all hyperpolarized protons are summarized in Table S-1 in the supporting information.

**Table 1:** Average signal enhancements of H^B^ and corresponding polarization % at different catalyst concentrations and solvent compositions, for PHIP-marker A and modified octreotide derivatives 1/2-A1 and 1/2-A3.

Sample		Signal enhancement and catalyst concentration										Solvent
		High 1.8 mM			Medium 0.9 mM			Low 0.45 mM				
**A**	Fmoc-Tyr(Prg)-OH	5654	± 590	45.3%	4738	± 1066	38.0%	4412	± 591	35.4%		MeOD-d_4_
**1-A1**	OctF1PrgAlc	1374	± 506	11.0%	488	± 191	3.9%	324	± 51	2.6%		MeOD-d_4_
		466	± 74	3.7%	–	–		–	–			52 vol% EtOD-d_6_/D_2_O
**1-A3**	OctF3PrgAlc	242	± 40	1.9%	66	± 19	0.5%	37	± 12	0.3%		MeOD-d_4_
**2-A1**	OctF1PrgAc	1251	± 237	10.0%	890	± 156	7.1%	364	± 58	2.9%		MeOD-d_4_
		896	± 152^a^	7.2%	822	± 130	6.6%	449	± 71	3.6%		50 vol% MeOD-d_4_/D_2_O
		–	–		–	–		815	± 129^b^	6.5%		36 vol% MeOD-d_4_/D_2_O
**2-A3**	OctF3PrgAc	731	± 114	5.9%	309	± 38	2.5%	184	± 21	1.5%		MeOD-d_4_

Averages for A are based on two measurements, and for the peptides on three measurements respectively.

^a^Concentrations of analyte and catalyst were reduced by a factor of 0.77, already considered in SE.

^b^Concentration of analyte was 1.2 mM, already considered in SE.

For regular measurements, the concentration of the respective peptide was chosen as 1 mM. The observed signal enhancements of over 1000 on average correspond to a proton polarization of 8%, leading to a theoretical reduction to about one millionth in measurement time and, taking the reaction time into account, still a 10,000-fold reduction in practice for a comparable signal-to-noise ratio. This allows detection of peptides at nanomolar concentrations in a single scan, if the sample is diluted right after the reaction. With our setup we were able to detect a hyperpolarized signal down to 20 µM peptide concentration in a single scan, owing to the limitations given by the reaction kinetics, the observation time window, and our spectrometers detection limit.

The measurements were initially conducted using methanol as both the catalyst and the peptide compounds are highly soluble in it. It is obvious that due to the toxicity of methanol for any in vivo application or cell culture water is the preferred solvent. Therefore, we reduced the amount of methanol in examined samples and carried out the measurements in solutions of 50 and 36 vol% of aqueous methanol.

With careful sample preparation, the concentration of methanol can be reduced to 10 vol%, which is well below the daily reference dose of 0.5 mg of methanol per kg body weight in a dose of a cancer diagnostics marker solution for an average human adult^54^. The biocompatibility can be improved by replacing the methanol with less toxic compounds, such as ethanol. 50 vol% aqueous ethanol solution was also PHIP active and led to SE of 466 in 1.8 M catalyst solution (Table 1). Experiments in neat water, however, were currently not feasible due to the insolubility of the utilized catalyst. If needed, water soluble catalysts for experiments in pure water are available and should be tested, some have been successfully applied to hydrogenate pyruvate^17^. On the other hand, a biphasic approach might help to circumvent this issue and separate the catalyst from the analyte. The later method has mostly been implemented in gas/liquid or gas/solid systems^55–57^.

The separation of the catalyst from the hyperpolarized peptide might be performed by the precipitation of the peptide, e.g., by mixing with an nonpolar solvent and dissolution in a clean solvent, as it was demonstrated for fumarate^25^.

The catalyst [1,4-bis-(diphenylphosphino)-butane]-(1,5-cyclooctadiene)rhodium(I) tetrafluoroborate was utilized in three concentrations: the lowest at 0.45 mM, a medium concentration of 0.9 mM and the highest at 1.8 mM.

**Figure 4:**
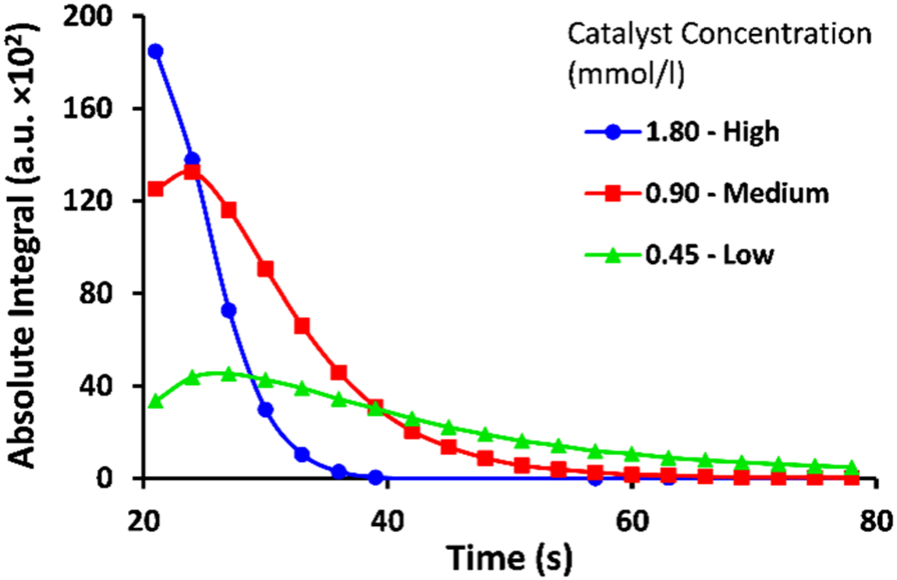
Signal intensities of the hyperpolarized protons during the reaction of 1 mM of the peptide F1PrgAc (**2-A1**) with parahydrogen at different catalyst concentrations. The reaction was started by bubbling parahydrogen for 15 s and then followed by a series of short acquisition pulses with 5° flip angles. At a low concentration of catalyst, the initial reaction rate is slow and the maximum in signal intensity is low but the polarization is maintained over a longer period of time. At a high catalyst concentration, the initial reaction rate and the maximum intensity are much higher and the maximum in signal intensity is reached immediately after bubbling.

These rather large amounts of catalyst were used to boost the reaction rate of the hydrogenation. When the label **A** was used solitarily in a PHIP experiment, the concentration of the catalyst was found to have almost no influence on the achievable SE factors. This indicates a fast binding and exchange of the substrate for this small molecule. However, for the investigated peptides large differences in the reaction rate were observed (Fig. 4).

With increased catalyst concentration, the initial reaction rate is higher, thus the maximum of the observable hyperpolarization is reached earlier. As the initial hyperpolarization had less time to decay, more of the hyperpolarized species are present at the moment of spectrum acquisition, which leads to a much larger observed signal intensity.

Due to the short T_1_ of proton spins, the hyperpolarization decays quickly after the reaction. Since the reaction rate is faster in the beginning and gradually slows down, the observable hyperpolarized signal goes proportionally through a maximum. As the reaction rate slows down, the hyperpolarization decays, and the observable signal intensity diminishes with increased reaction time.

At higher concentrations of catalyst, the signals of the product after the reaction show some broadening and changes in chemical shift. This might indicate the formation of an intermediate catalyst–product complex that decays slowly^58^. This complex, however, can be created by the catalyst binding not just to the desired alkyne moiety but also to other moieties such as aryl groups or the peptide backbone^28^. As those groups can compete with the PHIP marker, decreasing the observed reactivity of the catalyst in comparison to smaller molecules with less interfering groups, this might explain the observations made for **A** solitarily.

We investigated the possibility of a further reaction of the allyl moiety to an alkyl moiety, yielding a propyl group (Fig. 2). For neat **A**, conventional 1D spectra and 2D TOCSY proved that the reaction does not stop at the allyl product and proceeds further. However, the product of the second reaction for the peptides was never observable either in conventional 1D, or in the 2D TOCSY spectra (Figures S-9 to S-12 in the Supporting Information). When the hydrogenation was carried out for the allyl variants **2-B1** and **2-B3** separately, we could hardly see any PHIP signal of the peptide, which indicated that the rate for the second reaction step from an allyl to an alkyl moiety was much slower for the peptides than the first step from alkyne to allyl. We also ensured that the disulfide bond of the peptide is not cleaved by the hydrogenation reaction (see chapter 5 in the SI).

As a reference for the determination of the SE, the values for the signal integrals at thermal equilibrium were determined from 1D spectra of the allyl group bearing samples **2-B1** and **2-B3** at a 1 mM concentration without starting a reaction. The integrals of these spectra correspond to the integrals of the reaction products of **1/2-A1** and **1/2-A3**, assuming a complete reaction of the propargyl to the allyl moiety with no further reduction to a propyl group. Similarly, the enhancement factors for **A** were determined by using the signal intensities of **B** at thermal equilibrium at 1 mM concentration.

Table 1 summarizes the average SE obtained for H^B^ of the allyl group. The biggest enhancements were observed for the high concentration of catalyst and lower enhancement for the medium and low concentrations, as it was expected. In contrast to our expectations based on the general results, marker **A** shows an increase of signal enhancement with lower catalyst concentrations at 25 s reaction time (see Table S-1 in the SI). This can be explained by the fast reaction of the marker with hydrogen at high concentrations of catalyst, so that the lifetime of the initial hyperpolarization largely decays by the time the acquisition started. While at lower catalyst concentrations the slower reaction speed leads to a broader maximum in time, as seen in Fig. 4. When the experiments were repeated with a shorter reaction time of 15 s, much higher enhancement factors were achieved. Considering the margin of error, all three catalyst concentrations gave similar enhancement factors for the marker **A**. These large errors could have been caused by a variation in shim quality due to remaining gas bubbles in the solution at these short reaction times, subsequently leading to large differences in the observed signal intensities.

For the peptides, the highest average SE of 1374 was observed for OctF1PrgAlc (**1-A1**) at a high catalyst concentration and 25 s reaction time. An even higher SE of 2056 (Fig. 3) was achieved for **1-A1** at a slightly higher catalyst concentration of 1.96 mM. This is about half of the enhancement achieved with **A** at low catalyst concentration at 15 s and corresponds to a proton polarization of about 16%. Generally, the high concentration of catalyst allows the greatest observable average enhancements. The nature of the *C*-terminal group does not seem to have a big influence on the achievable signal enhancement with the marker at the *C*-terminal position, as the variants **1-A1** and **2-A1** both show similarly high enhancement factors at high and low catalyst concentrations. The difference is only at the medium catalyst concentration, where the enhancement for the carboxyl variant **2-A1** is 890, which is about twice as big when compared to the 488 of the alcoholic variant **1-A1**.

While the average enhancement values of 242 and 731 for the variants **1-A3** and **2-A3** with the PHIP marker at position 3 are still very good, they fall behind the variants **1-A1** and **2-A1** with the marker at position 1. This is probably due to the better accessibility of the triple bond when the PHIP-marker is placed at the end of the peptide chain close to the sterically less demanding disulfide bridge. Placing the marker in the middle of the chain most probably hinders the catalyst from binding to the triple bond, due to interference with neighboring groups^28^.

For the peptides with the attached marker at the position 3, the difference between the carboxyl and alcohol variants is more pronounced. The carboxyl variant achieves triple the enhancement of the alcohol variant at high catalyst concentrations and almost five times at medium or low catalyst concentrations. The reason for this difference is not known. It could be speculated that with the different *C*-termini there is a difference in the rigidity of the peptide due to different hydrogen bonding patterns in the beta-sheet of the peptides. Whether a more or less rigid structure of the peptide facilitates a faster reaction would be subject to further studies.

Using a solution of 50 vol% aqueous (aq.) methanol, we were able to reach enhancement factors of 800–900 at medium and high catalyst concentrations. At 36 vol% aq. methanol, only the low concentration of catalyst could be employed, due to solubility issues. With 1.2 mM concentration of the peptide **2-A1**, we were still able to achieve a signal enhancement of 815.

With 50 vol% ethanol, as a less toxic alternative to methanol, we achieved a signal enhancement of 466 for **1-A1**. Overall, the amount of alcohol in the solvent does not seem to have a big impact on the reaction rate, it rather influences the bubble formation and mixing behavior of hydrogen gas. By carefully tuning the reaction parameters such as hydrogen pressure and flow rate to the solvent composition, it should be possible to further enhance the mixing behavior and thus the reaction speed to reach even higher signal enhancements in aqueous solutions, comparable to those in pure methanol. For the reactions in neat water, a water-soluble catalyst is required. Alternatively, several methods have been developed to remove the organic solvent after the reaction and replace it with water, such as phase transfer or rapid evaporation by spray-flash distillation of the organic solvent. These treatments can additionally remove a catalyst from the solution^59,60^. These steps require more specialized equipment, but are essential to increase the biocompatibility of the hyperpolarized agents.

### Bioactivity by cell binding studies

**Figure 5:**
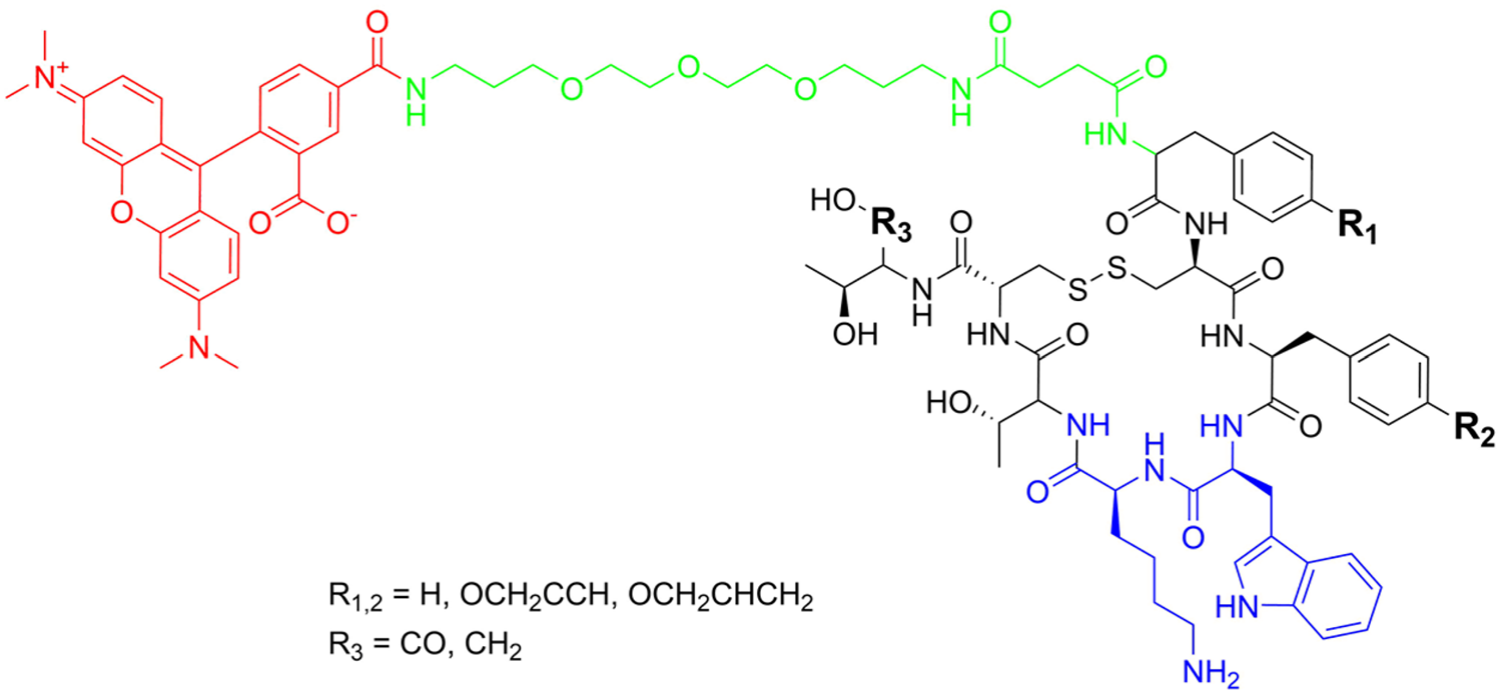
General structure of the peptides marked with TAMRA (red) conjugated by a linker structure (green). The linker was attached to the N terminus of the peptides to prevent possible interference of TAMRA with the main binding motif of the peptides (blue).

In order to check whether the modifications on the octreotide have an influence on its interaction with somatostatin receptors in living cells, a binding study on different human cell lines was performed. For the detection of the bound peptides, octreotide and the derivatives were further modified by attaching the fluorophore TAMRA to the *N* terminus. To prevent the fluorophore from interacting with the binding site of octreotide derivatives, a 4,7,10-trioxa-1,13-tridecanediamine succinate (TTDS) spacer was added between the peptide *N* terminus and the *N*-hydroxy succinimide (NHS)-activated fluorophore. Both, the linker and the fluorophore, were attached to the peptides by manual SPPS (Fig. 5).

Human lung cancer cells A549 and human embryonic kidney cells HEK293 were utilized for the binding studies. A549 are widely used in binding studies with somatostatin analogues^61,62^. Like other tumor cell types, they show high expression of a wide range of somatostatin receptors, including SSTR2, and have been tested with octreotide and derivatives to find promising drug candidates for tumor treatment^63,64^. HEK293 also show medium SSTR2 expression, which makes them suitable for evaluation of octreotide-based drugs^65,66^.

**Figure 6:**
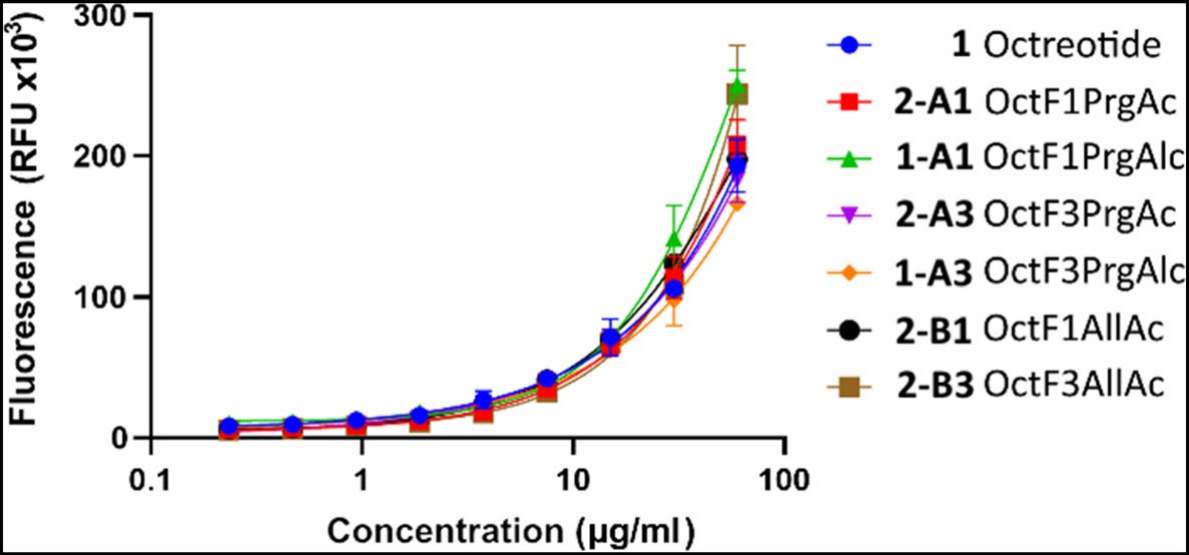
Fluorescence in Relative Fluorescence Units (RFU) of the TAMRA conjugated octreotide variants bound to the A549 cells against their concentration during incubation. The fluorescence for all variants rises almost equally with increased concentration indicating the binding of all variants to the cells with similar affinity.

Compared to the unmodified octreotide **1**, the investigated modifications hardly had an influence on the binding behavior (Fig. 6, Figure S-22). The allyl variants **2-B1** and **2-B3** are especially important, as they represent the active form of the compounds after hydrogenation and hyperpolarization. It has been shown that these, like all other variants, interact equally well with the cells surface.

The F1Prg variants seem to perform slightly better than the F3Prg ones. This could be attributed to the position of the PHIP marker, which is located away from the main binding motif Trp4-Lys5, thus causing less interference upon the interaction with the SSTRs. However, those differences are not significant, the allyl variant **2-B3** shows a higher fluorescence than **2-B1** at the highest concentration. Also, no significant difference was observed between the alcohol and carboxyl *C*-termini. At concentrations below 10 µg/ml, all variants behave very similarly. Experiments on binding only TAMRA to the cells showed an about two times reduced binding efficiency compared to the system TAMRA-linker-octreotide, confirming a specific binding of peptides to the SSTRs of the cells (Figures S-23 and S-24).

## Summary and conclusion

In this paper we present the hyperpolarization-driven signal enhancement of octreotide derivatives in the high field (500 MHz) proton NMR. We successfully synthesized biocompatible derivatives of octreotide, a cancer cell-targeting drug widely used in clinical applications, and proved their suitability for high signal enhancements. This makes them promising candidates for further studies in imaging applications of MR utilizing the PHIP hyperpolarization technique. This technique is fast, easy to use, requires only small investments in equipment, and is compatible with almost all standard NMR and MRI devices.

By careful tuning the reaction conditions of the hydrogenation process with parahydrogen and under precise timing with a fully automated PASADENA setup, average signal enhancement factors of up to 1400 and maximum enhancements of 2000 were reached. This major boost in sensitivity allows the detection of peptides down to the nanomolar regime and greatly reduces measurement times by factors of up to 100,000 in practice.

We demonstrated that incorporation of PHIP labels and the hydrogenation of such labels do not diminish the biological activity of the modified variants of octreotide. The binding to human lung cancer and kidney cell lines expressing somatostatin receptors remained largely unchanged. This provides a further proof for the ease of operation of the PHIP technique since no further modification of the peptide after hydrogenation is necessary.

The hydrogenation reactions could be performed in mixtures of water and very low, down to 36% and even 10%, amounts of methanol or mixtures of water and ethanol, which further contributes to the biocompatibility of this approach.

The T_1_ times of the hyperpolarized protons at our magnetic field strength of 11.75 T are in the order of 3–4 s, leading to fast relaxation of the polarized state. This limits the level of achievable polarization during a given reaction time and constraints the time-window of the detection of the hyperpolarization. An efficient way to widen the application window is the enrichment of the compound, close to the hydrogenated bond, with spins with a longer T_1_ time, such as ^13^C or ^15^N^67,68^, and the transfer of the hyperpolarization to those spins, employing pulse sequences such as PH-INEPT or ESOTHERIC, or by cycling the magnetic field strength to zero or ultra-low field (ZULF)^68–70^.

In order to achieve a first estimate on the effects of ^13^C-labelling, we performed high-field ^13^C-T_1_ relaxation time measurements at 11.7 T (note: clinical MRI scanners typically operate at 0.5–1.5 T) on the protonated monomer H-l-tyrosine(allyl)-OH (see SI Sect. 3.6). At this field, relaxation times in the range of 3–7 s for the allyl moiety and of 12–18 s for the quaternary carbons were observed for the allyl bearing marker **B**. Thus, a simple switch of the detection nucleus leads to triple the width of the application window compared to protons. Further improvements of the relaxation times are achievable by partial deuteration of the side chain, i.e., by replacing protons in the vicinity of the hydrogenated bond and the quaternary carbon with deuterium^67^ or possibly by selective double labelling for the phenyl ring in order to create a long-lived spin-state^71^.

With the maximum proton polarization of up to 16% achieved in this work at high field (11.7 T), we have laid a solid foundation for further experiments in reaching high carbon polarization. In the follow-up investigation, we plan to extend our studies also to lower magnetic fields of 1.5 T, similar to the current state of MRI scanners. We expect this to lead to even higher enhancements and, owing to the smaller chemical shift anisotropy (CSA) relaxation, most probably also to longer ^13^C-relaxaton times. To that end, we plan to synthesize marker molecules with ^13^C labeling on a quaternary carbon close to the unsaturated bond, such as, for example, an ester of propiolic acid or propargyl alcohol, partially deuterate the side chain^67^, and investigate their relaxation times as a function of the magnetic field.

Finally, the question concerning the possible application of the PHIP-labeled octreotide arises. Owing to the limited life-time of the hyperpolarization, only the experiments with fast enough (compared to the T_1_-relaxation) binding of the octreotide derivative to the target protein can profit from the hyperpolarization. The present T_1_ times of octreotide derivatives allow the application of our technique to mechanistic and kinetic studies of somatostatin binding to SSTRs in cell cultures or animal models and not in humans or big animals in vivo. The spectral separation of bound hyperpolarized octreotide from free dissolved states can be achieved either by monitoring the chemical shift or simply the NMR line-shape, since the line width is usually strongly increased for bound species in comparison to unbound (see Koenig et al.^72^, Kimmich et al.^73^ and Bodurka et al.^74^).

In order to make the hyperpolarization also usable for clinical application, such as, e.g., tumor diagnostics in an MRT scanner, a substantial prolongation of the relaxation times is necessary and could be achieved by the usage of long-lived spin states of carbon spins. This can provide new methods to investigate the kinetics of octreotide distribution in tumor tissue upon local administration.

## Methods

### Synthetic methods

#### Synthesis of Fmoc-Thr(tBu)-ol

Fmoc-Thr(tBu)-ol was obtained by reduction of Fmoc-Thr(tBu)-OH using NaBH_4_ in THF and water according to the procedure described by Rodriguez et al.^75^ To a solution of Fmoc and tertbutyl-protected threonine (1.5 g, 3.77 mmol) in THF (15 ml), cooled on an ice-salt bath, *N*-methyl morpholine (420 µl, 3.78 mmol) and isobutyl chloroformate (490 µl, 3.77 mmol) were successively added. After one minute a solution of sodium borohydride (580 mg, 15.33 mmol) in water (2.1 ml) was added at once, producing a strong evolution of gas, followed by water (200 ml) 30 s afterwards. The cloudy suspension was extracted with DCM two times. The organic phase was collected and evaporated in a rotary evaporator (40 °C, 450 mbar), yielding a clear viscous product.

#### Synthesis of Fmoc-TTDS spacer

The synthesis of Fmoc-TTDS was carried out following the procedure described by Zhao et al.^76^. To a solution of 11.02 g (50 mmol) 4,7,10-trioxa-1,13-tridecanediamine in 200 ml acetonitrile at 0 °C 5 g (50 mmol) succinic anhydride in 200 ml acetonitrile was added dropwise in the period of one hour. The resulting precipitate was collected and redissolved in 500 ml 50%(v/v) acetonitrile in water. To this a solution of 21.93 g (65 mmol) Fmoc-OSu in 250 ml acetonitrile was added over the course of one hour at a temperature of 0 °C. After adjusting the reaction mixture to pH 8 using DIEA, the mixture was warmed to room temperature and stirred overnight. The solvent mixture was removed under reduced pressure and replaced by 500 ml of concentrated, aqueous NaHCO3. This mixture was then washed three times with 250 ml ethyl acetate, acidified to a pH of 1 with concentrated muriatic acid and then extracted with again three times 250 ml ethyl acetate. The combined organic phases were dried over MgSO4 and the solvent was removed under reduced pressure to yield 17.3 g (32 mmol) of the product as a colorless oil.

#### Synthesis of TAMRA-NHS

The 5,6-TAMRA synthesis (an isomeric mixture of 5- and 6-TAMRA, from now on referred to as TAMRA) was based on Kvach et al.^77^ and was performed under argon on a Schlenk line.

In a typical synthesis, 4.56 g 3-dimethylaminophenol (33 mmol, 1 eq.) was dissolved in 90 ml dry toluene, and under stirring 7.65 g finely ground trimellitic anhydride (40 mmol, 1.2 eq.) was added. After 24 h reflux the mixture was cooled and the precipitate was washed at least 3 times with 20 ml cold toluene. The precipitate was then dissolved in 120 ml MeOH, which was removed afterwards by rotary evaporation to obtain a benzophenone intermediate with an average yield of 70%. Back under argon atmosphere, 7.5 g benzophenone (22 mmol, 1 eq.) was dissolved in 170 ml dry DMF, after that 3.94 g 3-dimethylaminophenol (28 mmol, 1.3 eq.) and 40 ml trimethylsilyl polyphosphate were added. The reaction mixture was refluxed for 3 h and cooled. After cooling, the solvent was removed under reduced pressure and the remaining residue was stirred overnight in 170 ml 5% NaOH at room temperature. The solution was diluted with 200 ml water and neutralized with concentrated HCl to precipitate the product. The product was washed with cold water and then with cold ether. The resulting isomeric mixture of TAMRA was used without further purification. An average yield of 1.4 g (15%) was obtained.

For NHS activation, 50 mg TAMRA (0.11 mmol, 1 eq.) was dissolved in 12 ml dry acetonitrile and cooled to 0 °C in an ice bath. 20.2 µl dry DIEA (0.11 mmol, 1 eq.) was added and right afterwards 17.9 µl DIC (0.11 mmol, 1 eq.) and 19 mg NHS (0.15 mmol, 1.3 eq.) were added. The mixture was stirred overnight in the ice bath, whereby the ice bath was allowed to warm up to room temperature overnight. Afterwards the acetonitrile was removed under reduced pressure. For further drying the product was freeze dried. A magenta-colored solid of the TAMRA-NHS ester was obtained and used without further purification. The obtained yield was around 29 g (50%). The MS chromatogram and LC ESI-mass spectra can be found in Figures S-3 and S-4.

#### SPPS

All peptides were synthesized manually by Fmoc-SPPS following Merrifield^50^, using the acid-labile 2-chlorotritylchloride (2-CTC) resin. Manual solid-phase amino acid incorporation and other solid-phase manipulations were carried out in polypropylene syringes fitted with a fritted polyethylene disk CEL-053, -1016 and -2020 purchased from Roland Vetter Laborbedarf OHG. Solvents and soluble reagents were added/removed by suction/pushing of the plunger. Solutions were agitated by shaking.

2 eq. of Fmoc-Cys(Trp)-OH were attached with the help of 2.2 eq. DIC and 2.2 eq. OxymaPure^®^ in DMF. All other amino acids were coupled using 2 eq of the Fmoc-protected amino acid, 4 eq. DIEA and 2 eq. HATU in DMF. Double coupling was employed for all amino acids. The Fmoc-TTDS spacer was coupled in the same way. For the attachment of TAMRA-NHS to the peptide chain or the first amino acid to the 2-CTC-resin only 4 eq. DIEA were added.

Cleaving of peptides from the solid support and removal of side chain protecting groups was achieved via acidolysis of the dry peptide-resin using a cleavage solution consisting of TFA, TIPS, anisole and H_2_O. The crude peptides were precipitated in cold MTBE and subsequently washed with MTBE and diethyl ether.

Cyclization of the peptides was done by the formation of intramolecular disulfide bridges following the procedure given by Sidorova et al.^51^. Linear peptides (1 eq.) were dissolved in methanol (0.3 mg/ml) and the pH of solutions was adjusted to be in the interval between 6.5 and 8.0, by adding highly diluted NH_3_ (aq.). Then few drops (2.7 eq.) of H_2_O_2_ were added. The reaction was left overnight and stopped by addition of few drops of acetic acid (99.5%). The solvents were evaporated and the solid products were dissolved in 50% ACN/H_2_O (v/v) and lyophilized. The peculiar details on the synthesis of each octreotide derivative can be found in the supplementary materials.

### NMR-methods

The parahydrogen enrichment was performed with a parahydrogen generator from Advanced Research Systems Inc. comprising a DE204A cryostat and an ARS 4HW compressor. The cryostat was cooled to 30 K. > 95% para-enriched hydrogen was delivered into the NMR sample tube placed inside the magnet at 7 bar pressures and room temperature. A custom-made setup was used for bubbling directly in the tube^32^.

The 5 mm screw-cap NMR sample tubes were ordered from Rototec Spintec (model 528-TR-7) and closed by a cap-adapter with an in- and outlet for the gases. A thin glass capillary was attached to the inlet and immersed in the sample. The bubbling process was synchronized with the NMR pulses and was stopped 2 s before the detection of NMR signals.

A standard PHIP sequence was applied by irradiation of a 45° pulse followed by the acquisition (see Figure S-6). All PHIP-spectra were recorded as “single-shot experiments”, collected as one scan. Full relaxation of the polarized protons in the products was observed at least 2 min after the hydrogenation.

## Supplementary Information

Supplementary Information.

## Data Availability

The sequence of octreotide is available in the PDB repository under accession number 1SOC^78^. All data generated or analysed during this study are included in this published article and its Supplementary Information files.
