# Supplementary material for: Parahydrogen-induced polarization allows 2000-fold signal enhancement in biologically active derivatives of the peptide-based drug octreotide
Source: Sci Rep. 2023 Apr 19;13:6388. doi: 10.1038/s41598-023-33577-2 (PMC10115808; doi:10.1038/s41598-023-33577-2)
Supplement: Supplementary file 1 — Supplementary Information. [file 41598_2023_33577_MOESM1_ESM.pdf]

Supporting Information

Parahydrogen-Induced Polarization Allows 2000-fold Signal Enhancement in Biologically Active Derivatives of the Peptide-Based Drug Octreotide

Jonas Lins,[a] Yuliya A. Miloslavina,[a] Stefania C. Carrara,[b] Lorenz Rösler,[a] Sarah Hofmann,[b] Kevin Herr,[a] Franziska Theiß,[a] Laura Wienands,[a] Olga Avrutina,[b] Harald Kolmar,*[b] and Gerd Buntkowsky*[a]

[a] Eduard-Zintl-Institute for Anorganic and Physical Chemistry, Technische Universität Darmstadt, Alarich-Weiss-Straße 8, 64287 Darmstadt, Germany
*E-Mail: gerd.buntkowsky@tu-darmstadt.de

[b] Clemens-Schöpf-Institute for Organic Chemistry and Biochemistry, Technische Universität Darmstadt, Alarich-Weiss-Straße 4, 64287 Darmstadt, Germany
*E-Mail: harald.kolmar@tu-darmstadt.de

Table of Contents

1. GENERAL INFORMATION 4
CHEMICALS 4
REVERSED PHASE HIGH-PERFORMANCE LIQUID CHROMATOGRAPHY (RP-HPLC) 4
ELECTROSPRAY MASS SPECTROMETRY (ESI-MS) 4
FLASH CHROMATOGRAPHY 4
2. SYNTHETIC METHODS 4
2.1.1. SYNTHESIS OF FMOC-THR(TBU)-OL 4
2.1.2. SYNTHESIS OF FMOC-TTDS SPACER 5
2.1.3. SYNTHESIS OF TAMRA-NHS 6
2.2. SPPS 7
2.2.1. RE- AND PREACTIVATION OF 2-CTC RESIN 7
2.2.2. COUPLING OF FMOC-THR(TBU)-OH TO 2-CTC RESIN 8
2.2.3. COUPLING OF FMOC-THR(TBU)-OL TO 2-CTC RESIN 8
2.2.4. COUPLING OF AMINOACIDS (PEPTIDE CHAIN ELONGATION) 8
2.2.5. COUPLING OF THE FMOC-TTDS SPACER 8
2.2.6. COUPLING OF TAMRA 8
2.2.7. CLEAVAGE OF PEPTIDES FROM RESIN, DEPROTECTION AND RECOVERY 8
2.2.8. CYCLIZATION OF LINEAR PEPTIDES 10
2.3. DETAILED SYNTHESIS OF OCTREOTIDE AND DERIVATIVES 10
2.3.1. SYNTHESIS OF FMOC-TRP-LYS-THR-CYS-THR-OH 10
2.3.2. SYNTHESIS OF OCTF3PRGAC (6) 10
2.3.3. SYNTHESIS OF OCTF1PRGAC (2-A1) 10
2.3.4. SYNTHESIS OF FMOC-TRP-LYS-THR-CYS-THR-OH FOR ALLYL VARIANTS (2-B1 AND 2-B3) 11
2.3.5. SYNTHESIS OF OCTF3ALLYLAC (2-B3) 11
2.3.6. SYNTHESIS OF OCTF1ALLYLAC (2-B1) 11
2.3.7. SYNTHESIS OF OCTF1PRGALC (1-A1) 11
2.3.8. SYNTHESIS OF OCTF3PRGALC (1-A3) 12
2.4. SYNTHESIS OF TAMRA CONJUGATED OCTREOTIDE AND DERIVATIVES 12
2.4.1. SYNTHESIS OF OCTF3PRGAC-TAMRA 12
2.4.2. SYNTHESIS OF OCTF1PRGAC-TAMRA 12
2.4.3. SYNTHESIS OF OCTF3ALLYLAC-TAMRA 12
2.4.4. SYNTHESIS OF OCTF1ALLYLAC-TAMRA 12
2.4.5. SYNTHESIS OF FMOC-TRP-LYS-THR-CYS-THR-2-CTC FOR CONJUGATION WITH FLUOROPHORE 13
2.4.6. SYNTHESIS OF OCTF3PRGALC-TAMRA 13
2.4.7. SYNTHESIS OF OCTF1PRGALC-TAMRA 13
2.4.8. SYNTHESIS OF OCTREOTIDE-TAMRA 13
3. NMR METHODS 14

3.1. PHIP EXPERIMENTS ... 14
3.1.1. ENHANCEMENT FACTORS ... 14
3.2. TIME SAVINGS ... 16
3.3. KINETIC MEASUREMENTS ... 16
3.4. 2D-TOCSY EXPERIMENTS ... 16
3.5. ^{1}H T_1 MEASUREMENTS ... 19
3.6. ^{13}C T_1-MEASUREMENTS ... 20
4. CELL BINDING ASSAY ... 22
5. STABILITY OF THE DISULFIDE BOND AFTER HYDROGENATION ... 24
6. RP-HPLC CHROMATOGRAMS ... 26
REFERENCES ... 33

1. General Information

Chemicals

Fmoc-protected amino-acids, 2-chlorotrityl chloride (2-CTC)-resin and ethyl cyano(hydroxyamino)acetate (Oxyma Pure®) were purchased from Iris Biotech GmbH. Acetylchloride, dichloromethane (DCM), diethyl ether, methyl *tert*-butyl ether (MTBE), triisopropylsilane (TIPS) and [1,4-bis-(diphenylphosphino)-butane]-(1,5-cyclooctadiene)-rhodium(I) tetrafluoroborate ([Rh(dppb)(COD)]BF_4) were purchased from Sigma-Aldrich. Isobutyl-chloroformate and *N*-methylmorpholin (NMM) were purchased from Acros Organics. Piperidine, NaOH, NH_3 solution (30-33%), *N,N*-Dimethylformamide (DMF), 1-[bis(dimethylamino)methylene]-1*H*-1,2,3-triazolo[4,5-*b*]pyridinium 3-oxide hexafluorophosphate (HATU), diisopropylethylamin (DIEA), anisole, *N,N'*-diisopropylcarbodiimid (DIC), tetrahydrofuran (THF), diethylether, trifluoroacetic acid (TFA), acetic acid, silica gel 60 and H_2O_2 were purchased from Carl Roth GmbH + Co. KG.

Reversed Phase High-Performance Liquid Chromatography (RP-HPLC)

Reversed phase high-performance liquid chromatography (RP-HPLC) for analytical purposes was conducted using a Waters HPLC setup consisting of a Waters Alliance e2695 equipped with a Waters 2998 PDA detector. The detection wavelength was chosen depending on the analyte between 214, 254, 280 and 301 nm. The eluent system for the HPLC system comprised eluent A (0.1% aq. TFA) and eluent B (99.9% acetonitrile and 0.1% TFA). Unless otherwise specified, analytical HPLC runs were conducted at a flow rate of 1 ml/min with a t gradient of 20% to 80% of eluent B over 20 min. For the analysis, a Nucleosil 100-5 C18 column from Macherey-Nagel (5 µm, 100 Å) was used. Preparative isolation of the peptide was performed on a Knauer Multokrom RP18 column 20×250 mm (5 µm, 100 Å) employing a flow rate of 9 ml/min and an isocratic elution, namely,40 % ACN in 0.1 % aqueous TFA over the course of 60 min.

Electrospray Mass Spectrometry (ESI-MS)

Electron Spray Ionization (ESI) mass spectra were recorded with a Bruker Impact II mass spectrometer.

Flash Chromatography

Flash chromatography was conducted on a Büchi Pure C815 Flash using hexane (A) and ethyl acetate (B) as solvents and a Büchi FlashPure Select 12 g Silica 15 µm column. The raw product was loaded by solid loading on silica. For this the substrate was dissolved in DCM, mixed with silica gel 60 and the solvent evaporated under reduced pressure.

2. Synthetic Methods

2.1.1. Synthesis of Fmoc-Thr(tBu)-ol

Fmoc-Thr(tBu)-ol was obtained by reduction of threonine Fmoc-Thr(tBu)-OH using $NaBH_4$ in an organic/aqueous medium according to the procedure described by Rodriguez *et al.*[1]
To a solution of Fmoc and tertbutyl-protected threonine (1.5 g, 3.77 mmol) in THF, (15 ml), cooled on an ice-salt bath, N-methyl morpholine (420 µl, 3.78 mmol) and isobutyl chloroformate (490 µl, 3.77 mmol) were successively added. After one minute a solution of sodium borohydride (580 mg, 15.33 mmol) in water (2.1 ml) was added at once, producing a strong evolution of gas, followed by water (200 ml) 30 seconds afterwards. The cloudy suspension was extracted with DCM two times. Centrifugation was used to aid the phase separation. The organic phase was collected and evaporated in a rotary evaporator (40 °C, 450 mbar), yielding a clear viscous raw product (1.47 g for the first, and 1.67 g for the second batch). The first batch was used without further treatment.
Cleanup of the second batch's raw product was performed by flash chromatography (1 minute at 8 % B in 92 % A, 16 minutes ramp up to 100 % B, 13 minutes B, t_R = 8 to 12 minutes). The fractions containing the product were united and evaporated. A clear viscous product was obtained (1.26 mg, 3.27 mmol).
Yield: 87 %; **RP-HPLC:** t_R = 19.6 min. **HRMS (ESI):** m/z calcd for $C_{23}H_{29}NO_4$: 384.22 $[M+H]^+$; found: 384.22, 328.12 $[M+H-(tBu)]^+$; found: 328.15.

2.1.2. Synthesis of Fmoc-TTDS Spacer

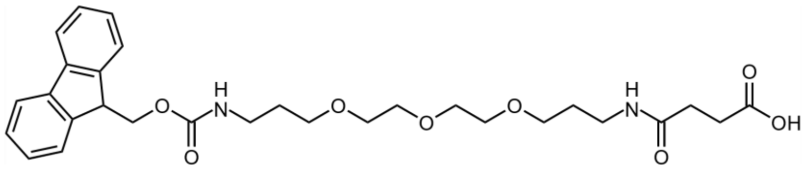

The synthesis of Fmoc-TTDS was carried out following the procedure described by Z. G. Zhao et al.[2] A solution of 11.02 g (50 mmol) 4,7,10-trioxa-1,13-tridecanediamine in 200 ml acetonitrile was cooled to 0 °C while adding 5 g (50 mmol) of succinic anhydride in 200 ml acetonitrile dropwise in the period of one hour. The formed precipitate was separated from the supernatant solution and redissolved in 500 ml of 50 % (v/v) acetonitrile in water. Again, under cooling to a temperature of 0 °C, a solution of 21.93 g (65 mmol) Fmoc-OSu in 250 ml acetonitrile was added to the mixture over the course of one hour. After adjusting the reaction mixture to pH = 8 using DIEA, the mixture was warmed to room temperature and stirred overnight. The solvent mixture was removed under reduced pressure and replaced by 500 ml of concentrated, aqueous $NaHCO_3$. This mixture was then washed three times with 250 ml ethyl acetate, acidified to a pH of 1 with concentrated muriatic acid and then extracted with again three times 250 ml ethyl acetate. The combined organic phases were dried over $MgSO_4$ and the solvent was removed under reduced pressure to yield 17.3 g (32 mmol) of the product as a colorless oil.

Yield: 64 %; **^{1}H NMR (500 MHz, $CDCl_3$):** δ 7.73 (d, J = 7.5 Hz, 2H), 7.58 (d, J = 7.7 Hz, 2H), 7.37 (t, J = 7.5 Hz, 2H), 7.28 (t, J = 7.4 Hz, 2H), 6.77 (t, J = 5.0 Hz, 1H), 5.52 (t, J = 6.0 Hz, 1H), 4.37 (d, J = 7.0 Hz, 2H), 4.19 (t, J = 7.0 Hz, 1H), 3.66 – 3.46 (m, 8H), 3.38 – 3.14 (m, 4H), 2.63 (t, J = 6.8 Hz, 2H), 2.45 (t, J = 6.8 Hz, 2H), 1.73 (dp, J = 18.1, 6.2 Hz, 4H); **^{13}C NMR (126 MHz, $CDCl_3$):** δ 175.24, 172.51, 156.75, 144.07, 141.36, 127.72, 127.10, 125.12, 120.00, 71.05 – 69.74 (m), 69.34, 66.51, 47.37, 38.93, 38.23, 30.92, 30.09, 29.48, 28.71.

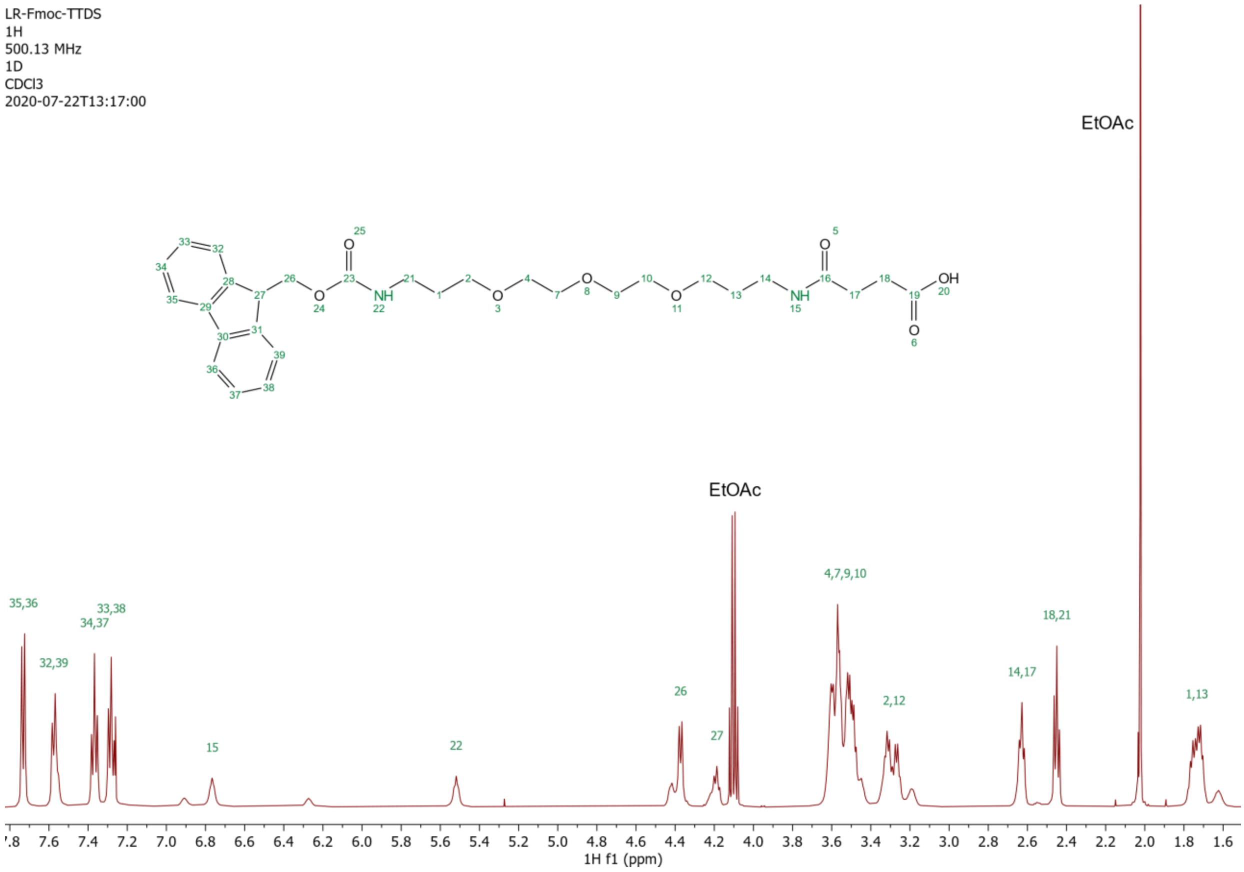

Figure S-1: ^{1}H NMR spectrum of Fmoc-TTDS.

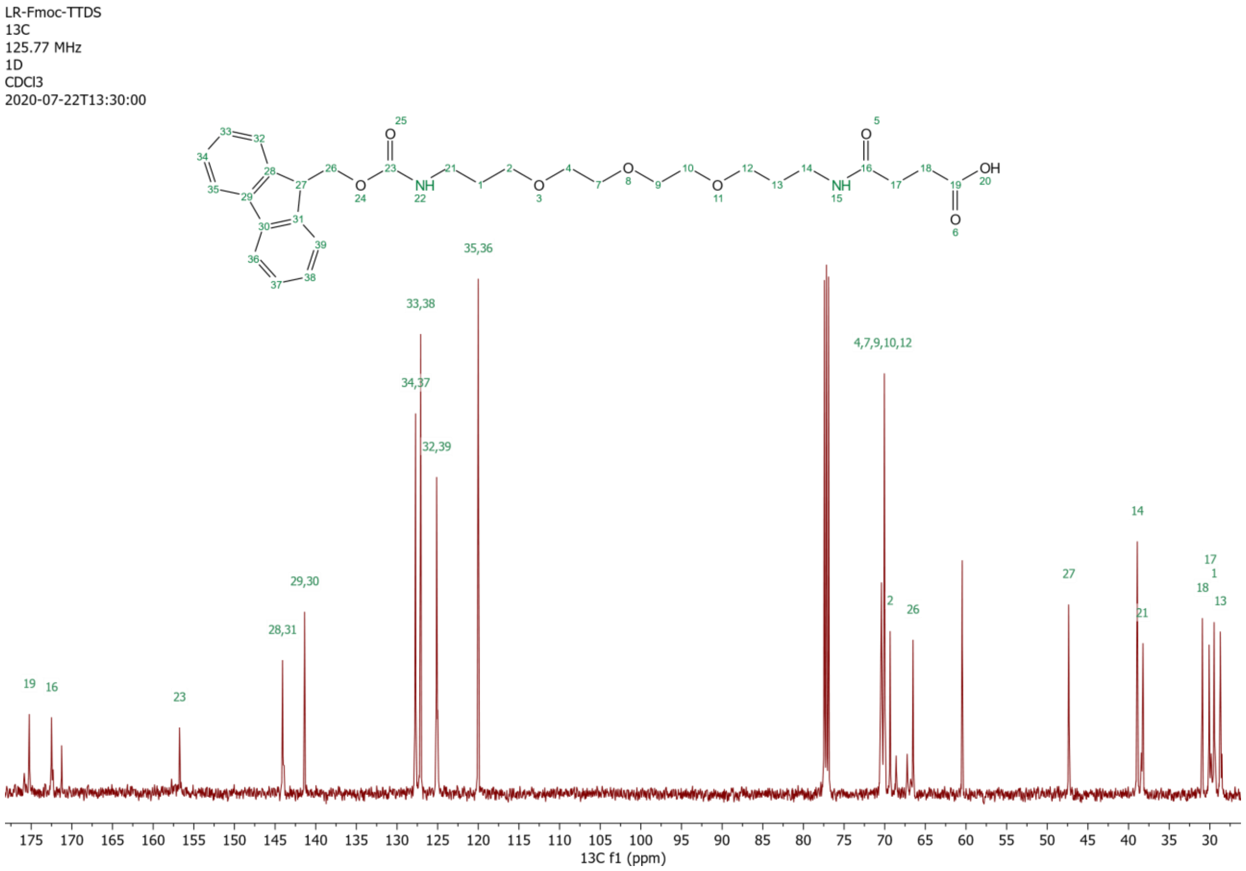

Figure S-2: ^{13}C NMR spectrum of Fmoc-TTDS.

2.1.3. *Synthesis of TAMRA-NHS*

5,6-TAMRA synthesis (an isomeric mixture of 5- and 6-TAMRA, from now on referred to as TAMRA) was based on Kvach *et.al.*[3] The procedure was performed under argon on a Schlenk line.

In a typical synthesis 4.56 g 3-dimethylaminophenol (33 mmol, 1 eq.) was dissolved in 90 ml dry toluene and 7.65 g finely ground trimellitic anhydride (40 mmol, 1.2 eq.) was added under stirring. After 24 h reflux the mixture was cooled and the precipitate was washed at least 3 times with 20 ml cold toluene. The precipitate was then dissolved in 120 ml MeOH and removed by rotary evaporation to obtain a benzophenone intermediate with an average yield of 70 %. Under argon atmosphere again, 7.5 g benzophenone (22 mmol, 1 eq.) was dissolved in 170 ml dry DMF and 3.94 g 3-dimethylaminophenol (28 mmol, 1.3 eq.) and 40 ml trimethylsilyl polyphosphate were added. The reaction mixture was refluxed for 3 h. After cooling, the solvent was removed under reduced pressure and the remaining residue was stirred overnight in 170 ml 5 % NaOH at room temperature. The solution was diluted with 200 ml water and neutralized with concentrated HCl to precipitate the product. The product was washed with cold water and cold ether. An isomeric mixture of TAMRA was obtained which was used without further purification. An average yield of 1.4 g (15 %) was obtained.

For NHS activation, typically 50 mg TAMRA (0.11 mmol, 1 eq.) was dissolved in 12 ml dry acetonitrile and cooled to 0 °C in an ice bath. 20.2 µl dry DIEA (0.11 mmol, 1 eq.) was added and afterwards 17.9 µl DIC (0.11 mmol, 1 eq.) and 19 mg NHS (0.15 mmol, 1.3 eq.) were added. The mixture was stirred overnight in the ice bath, whereby the ice bath was allowed to come to room temperature overnight. Afterwards the acetonitrile was removed under reduced pressure. For further drying the product was freeze dried. A pink solid of the TAMRA-NHS ester was obtained and used without further purification. An average yield of 29 g (50 %) was obtained.

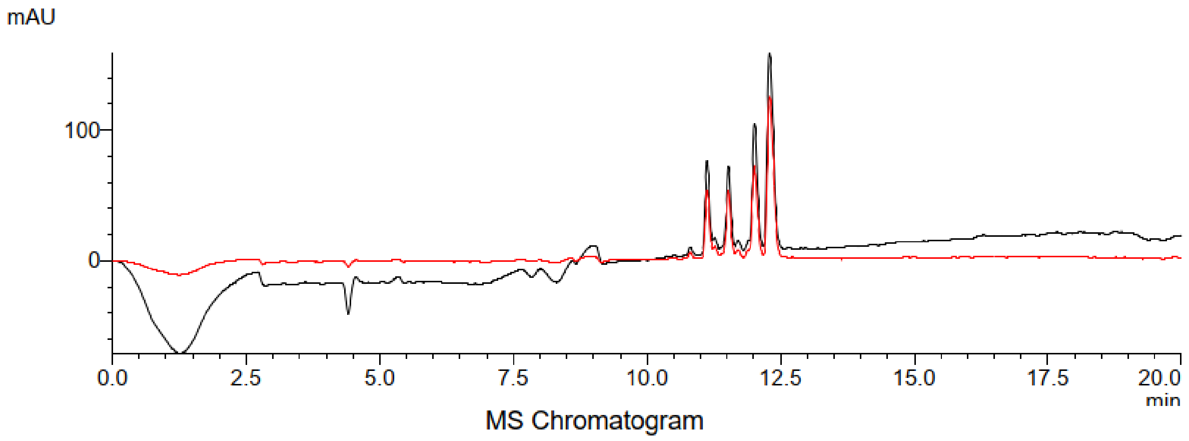

Figure S-3: Chromatogram acquired during LC-ESI-MS of activated TAMRA-NHS ester using water with 0.1 % (v/v) formic acid (eluent A) and acetonitrile with 0.1 % (v/v) formic acid (eluent B) with a gradient of 10 to 100 % B. Black trace: 220 nm; red trace: 280 nm; educt (TAMRA) t_R = 11.1 – 11.7 min.; product (TAMRA-NHS) t_R = 11.8 – 12.6 min.

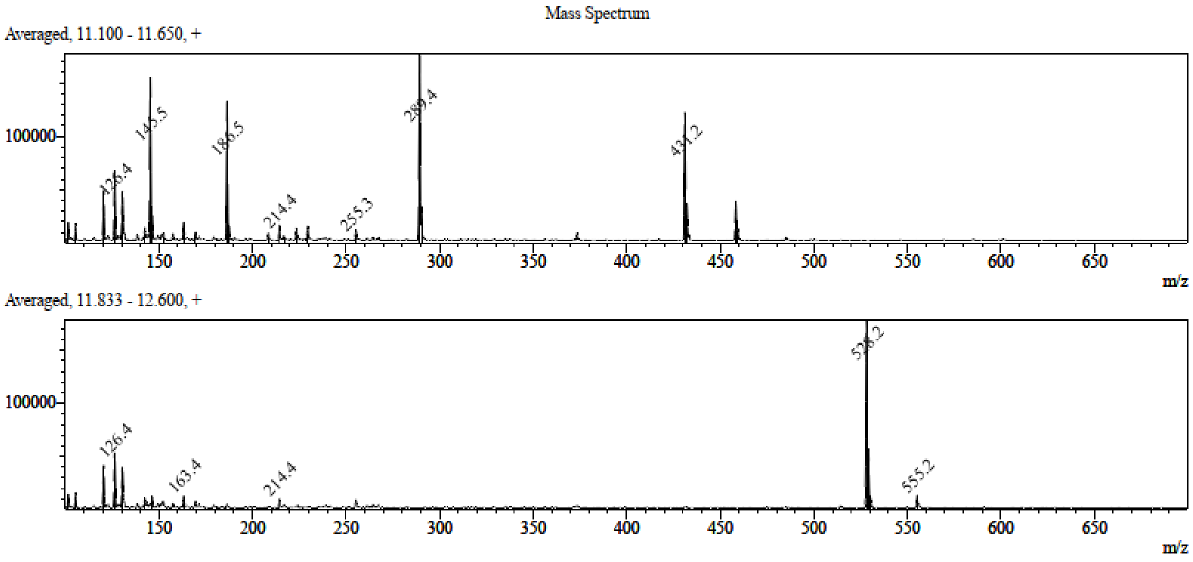

Figure S-4: LC-ESI-Mass spectra of TAMRA (averaged over 11.10 - 11.65 min.) and activated TAMRA-NHS ester (averaged over 11.83 – 12.60 min.)

2.2. SPPS

All peptides were synthesized manually by Fmoc-SPPS according to Merrifield,[4] using the acid-labile 2-chlorotritylchloride (2-CTC) resin. Manual solid-phase amino acid incorporation and other solid-phase manipulations were carried out in polypropylene syringes fitted with a fritted polyethylene disk CEL-053, -1016 and -2020 purchased from Roland Vetter Laborbedarf OHG. Solvents and soluble reagents were added/removed by suction/pushing of the plunger. Solutions were agitated by shaking.

2.2.1. Re- and Preactivation of 2-CTC Resin

2-CTC resin is used for the immobilization of the peptide during SPPS. Due to air humidity the resin can lose some of its activity during storage. Therefore, a reactivation of the resin following the procedure given by García-Martín *et al*.[5] can be advantageous. For the reactivation of the resin, the 2-CTC resin was lyophilized overnight to remove any moisture. Then 10 vol.-% acetyl chloride in DCM was added and shaken for one hour, washed three times with DCM and three times with DMF. Then for the preactivation of the resin 0.5 ml DIEA in 3.5 ml DMF were added and shaken for 30 minutes, leading to a change in color of the resin from yellow to deep red. After removal of the solution, the preactivated resin was washed three times with DMF.

2.2.2. *Coupling of Fmoc-Thr(tBu)-OH to 2-CTC Resin*

Fmoc-Thr(tBu)-OH (2 eq., 0.4 M) in DMF was coupled to 2-CTC resin using DIEA (4 eq.) as a base. The mixture was shaken for approx. 30 minutes. The solution was removed and the resin was washed four times with DMF. The coupling procedure was repeated a second time and the resin was washed again four times with DMF. The Fmoc group was cleaved with 20 vol.-% piperidine in DMF (1x5 min, 1×15 min) and the resin was washed 6 times with DMF. The supernatant solution of the Fmoc cleavage was collected and topped up with 20 vol.-% piperidine in DMF to 20 ml in a volumetric flask. A dilution series (10-, 100-, 1000-fold) was prepared in triplicate and the absorption at 301 nm was measured in a UV/Vis spectrometer to determine the loading of the resin. Depending on the concentration of the cleaved Fmoc, either the 100-fold or the 1000-fold diluted samples were measured.

2.2.3. *Coupling of Fmoc-Thr(tBu)-ol to 2-CTC Resin*

a)
Following a synthesis by Wenschuh *et al.*[6] for coupling amino alcohols to 2-CTC resin, 1.4 g Fmoc-Thr(tBu)-ol (3.8 mmol, 3.25 eq.), obtained in 2.1.1 (batch 1), and 614 µl pyridine (7.6 mmol) in 9 ml DCM were added to 0.8 g 2-CTC-resin (1.46 mmol/g, 1.17 mmol) and shaken for about 72 h at room temperature. Subsequently, the solution was removed and the resin was washed three times with DCM. The Fmoc group was cleaved with 20 vol.-% piperidine in DMF (1×5 min, 1×15 min) and the resin was washed 6 times with DMF. The solution of the Fmoc cleaving was collected and filled up with 20 % piperidine in DMF to 20 ml in a volumetric flask. A dilution series (10-, 100-, 1000-fold) was prepared in triplicate and the absorption at 301 nm was measured in a UV/Vis spectrometer to determine the loading of the resin. Depending on the concentration of the cleaved Fmoc, either the 100-fold or the 1000-fold diluted samples were measured.

b)
556 mg of Fmoc-Thr(tBu)-ol (1.45 mmol, 2.48 eq.), obtained in 2.1.1 (batch 2), in 4 ml DCM were coupled to 0.4 g 2-CTC-resin (1.46 mmol/g, 0.58 mmol) using 0.57 ml DIEA (3.28 mmol, 5.6 eq.) as base. The mixture was shaken for approx. 72 hours. Thereupon, the solution was removed and the resin was washed three times with DCM. The Fmoc-group was removed as described above.

2.2.4. *Coupling of Aminoacids (Peptide Chain Elongation)*

Fmoc-Cys(Trp)-OH was attached using 2 eq. of the Fmoc protected amino acid, 2.2 eq. of DIC and 2.2 eq. of OxymaPure® in DMF. All other amino acids were attached by employing 2 eq. of the corresponding Fmoc-protected amino acid, 2 eq. of HATU and 4 eq. of DIEA in DMF. The reaction vessel was shaken for half an hour at room temperature. All amino acids were attached by double coupling, after which the Fmoc group was removed with 20 vol.-% piperidine in DMF (1×5 min, 1×15 min) and the resin was washed 6 times with DMF.

2.2.5. *Coupling of the Fmoc-TTDS Spacer*

Attachment of the spacer was performed in the same way (2 eq. +2 eq. HATU +4 eq. DIEA) as the attachment of the amino acids.

2.2.6. *Coupling of TAMRA*

For the attachment of the dye the TAMRA-NHS ester obtained in 2.1.3 was used. The reaction was performed similarly to the attachment of the amino acids with 4 eq. of DIEA as base, but no activation agent was needed and only a single coupling step was employed. The reaction was carried out overnight. After removing the solution, the resin was washed with DMF until the supernatant showed no more signs of coloration.

2.2.7. *Cleavage of Peptides from Resin, Deprotection and Recovery*

Cleaving of peptides from the solid support and removal of side chain protecting groups was achieved via acidolysis of the dry peptide-resin using a cleavage solution consisting of TFA/TIPS/anisole/H_2O (47:1:1:1, v:v:v:v). The reaction mixture was shaken for 1-2 h at room temperature before filtering and precipitation in cold MTBE and subsequent

washing with MTBE and diethyl ether to yield the crude unprotected peptides. Separation of the precipitated peptides from the supernatant solutions was achieved by centrifugation.

2.2.8. Cyclization of Linear Peptides

Cyclization of the peptides was done by the formation of intramolecular disulfide bridges following the procedure given by Sidorova *et al*.[7] The linear peptides were dissolved in methanol with a peptide concentration of 0.3 mg/ml. The pH was adjusted between 6.5 and 8.0 by adding highly diluted $NH_{3\ (aq.)}$. Then, 2.7 eq. of H_2O_2 were added. The reaction was left to react overnight. The reaction was stopped by addition of a few drops of acetic acid (99.5 %). The solvents were evaporated and the solid product was dissolved in 50 % ACN/H_2O (v/v) and lyophilized. The cyclization was monitored by RP-HPLC and ESI mass spectrometry.

2.3. Detailed Synthesis of Octreotide and Derivatives

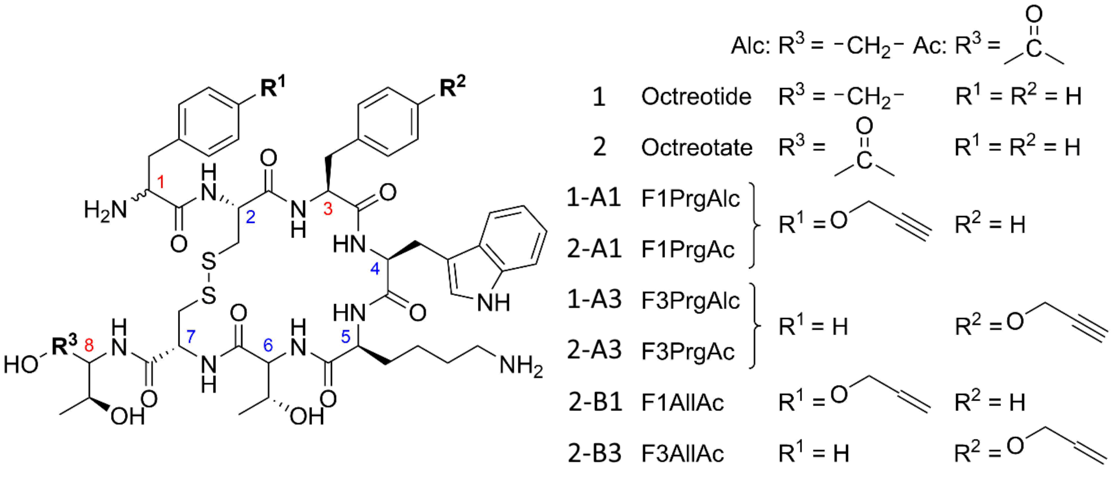

Figure S-5: Structures of the octreotide variants with incorporated PHIP labels and hydration products.

2.3.1. Synthesis of Fmoc-Trp-Lys-Thr-Cys-Thr-OH

For the precursor of the acid variants (**2-A1** and **2-A3**) 1.04 g (max. load: 1.46 mmol/g, 1.52 mmol) 2-CTC resin was preactivated according to 2.2.1 for 2 h. The resin was loaded with Fmoc-Thr(tBu)-OH according to 2.2.2. (load: 1.45 mmol). The amino acids cysteine, threonine, lysine and tryptophan were coupled according to 2.2.4.; 2.84 g of loaded resin were obtained and split up for the synthesis of different variants.

2.3.2. Synthesis of OctF3PrgAc (6)

0.96 g (0.49 mmol) of the loaded resin from 2.3.1 were coupled with the amino acids propargyl tyrosine, cysteine and D-phenylalanine according to 2.2.4. to yield 1.43 g of loaded resin. 0.51 g (0.175 mmol) were used for cleaving with TFA according to 2.2.7. 206 mg (0.189 mmol) of crude linear peptide were obtained.
RP-HPLC: t_R = 10.6 min.
HRMS (ESI): m/z calcd for $C_{52}H_{68}N_{10}O_{12}S_2$: 1089.45 $[M+H]^+$; found = 1089.45, 545.23 $[M+2H]^{2+}$; found = 545.23.
54.5 mg (0.050 mmol) were used in cyclization test reactions similar to 2.2.8, but with varying peptide concentrations or water as solvent, to yield 35.1 mg (0.032 mmol, 64 % cyclization yield) of cyclic peptide. Also, 109 mg (0.1 mmol) of the linear peptide were cyclized in another reaction according to 2.2.8 and yielded 92.4 mg (0.085 mmol, 85 % cyclization yield) of cyclic peptide. The fractions of cyclic peptide were united for an overall yield of 127 mg (0.117 mmol).
RP-HPLC: t_R = 10.0 min. **HRMS (ESI):** m/z calcd for $C_{52}H_{66}N_{10}O_{12}S_2$: 1087.44 $[M+H]^+$; found = 1087.44, 544.22 $[M+2H]^{2+}$; found = 544.22.

2.3.3. Synthesis of OctF1PrgAc (2-A1)

1.89 g (0.965 mmol) of loaded resin obtained from 2.3.1 were coupled with phenylalanine and cysteine to yield 2.34 g loaded resin. 1.17 g (0.483 mmol) of this loaded resin were coupled with propargyl tyrosine according to 2.2.4 to yield 1.34 g of loaded resin. 0.52 g (0.187 mmol) of this loaded resin were used for cleavage with TFA according to 2.2.7.; 197.5 mg (0.181 mmol, 97 % cleaving yield) of linear peptide were obtained.

RP-HPLC: t_R = 10.5 min. **HRMS (ESI):** m/z calcd for $C_{52}H_{68}N_{10}O_{12}S_2$: 1089.45 $[M+H]^+$; found = 1089.45, 545.23 $[M+2H]^{2+}$; found = 545.23.
94.5 mg (0.087 mmol) of linear peptide were cyclized according to 2.2.8 to yield 76.7 mg (0.071 mmol, 82 % cyclization yield) of cyclized peptide.
RP-HPLC: t_R = 11.0 min. **HRMS (ESI):** m/z calcd for $C_{52}H_{66}N_{10}O_{12}S_2$: 1087.44 $[M+H]^+$; found = 1087.43, 544.22 $[M+2H]^{2+}$; found = 544.22.

2.3.4. Synthesis of Fmoc-Trp-Lys-Thr-Cys-Thr-OH for Allyl Variants (2-B1 and 2-B3)

0.3 g 2-CTC resin (1.46 mmol/g, 0.44 mmol) were preactivated according to 2.2.1 and loaded with Fmoc-Thr(tBu)-OH, in a single coupling reaction for 1 h (load: 1.36 mmol/g, 0.408 mmol). The amino acids cysteine, threonine, lysine and tryptophan were coupled according to 2.2.4. 0.82 g of loaded resin were obtained and split for the following syntheses.

2.3.5. Synthesis of OctF3AllylAc (2-B3)

0.41 g (0.204 mmol) of loaded resin obtained from 2.3.4 were coupled with propargyl tyrosine, cysteine and D-phenylalanine according to 2.2.4. to yield 0.47 g of loaded resin. 238 mg (0.103 mmol) of the loaded resin were used for coupling with a dye (2.4.3). The rest (234 mg, 0.102 mmol) was cleaved with TFA according to 2.2.7 to yield 97.1 mg (0.089 mmol) of linear peptide.
RP-HPLC: t_R =11.2 min. **HRMS (ESI):** m/z calcd for $C_{52}H_{70}N_{10}O_{12}S_2$: 1091.47 $[M+H]^+$; found = 1091.47, 546.24 $[M+2H]^{2+}$; found = 546.24.
After cyclization according to 2.2.8 90.8 mg (0.083 mmol) of cyclized peptide were obtained.
RP-HPLC: t_R = 10.4 min. **HRMS (ESI):** m/z calcd for $C_{52}H_{68}N_{10}O_{12}S_2$: 1089.45 $[M+H]^+$; found = 1089.45, 545.23 $[M+2H]^{2+}$; found = 545.23.

2.3.6. Synthesis of OctF1AllylAc (2-B1)

0.4 g (0.199 mmol) of loaded resin obtained from 2.3.4 were coupled with phenylalanine, cysteine and propargyl tyrosine according to 2.2.4. to yield 0.46 g of loaded resin. 234 mg (0.101 mmol) of the loaded resin were used for coupling with a dye (2.4.4). The rest (227 mg, 0.098 mmol) were cleaved with TFA according to 2.2.7 to yield 91.6 mg (0.084 mmol) of linear peptide.
RP-HPLC: t_R = 11.0 min. **HRMS (ESI):** m/z calcd for $C_{52}H_{70}N_{10}O_{12}S_2$: 1091.47 $[M+H]^+$; found = 1091.47, 546.24 $[M+2H]^{2+}$; found = 546.24.
After cyclization 98.4 mg (0.090 mmol) of cyclized peptide were obtained, according to 2.2.8.
RP-HPLC: t_R = 11.6 min. **HRMS (ESI):** m/z calcd for $C_{52}H_{68}N_{10}O_{12}S_2$: 1089.45 $[M+H]^+$; found = 1089.45, 545.23 $[M+2H]^{2+}$; found = 545.23.

2.3.7. Synthesis of OctF1PrgAlc (1-A1)

For the Synthesis of OctF1PrgAlc 0.8 g of 2-CTC resin (max. load: 1.46 mmol/g, 1.17 mmol) were weighted into a syringe with a polyethylene filter. 1.4 g (3.8 mmol, 3.25 eq.) of Fmoc-Thr(tBu)-ol were coupled according to 2.2.3 a). After cleaving of the Fmoc protecting group the initial load was determined by UV/Vis spectroscopy to be 0.29 mmol/g (0.23 mmol, yield of 19.7 %).
Coupling of the Fmoc protected amino acids Thr, Cys, Thr, Lys, Trp, Tyr(Prg), Cys and D-Phe was performed according to 2.2.4. After cleaving of the peptide from the resin according to 2.2.8, a crude yield of 238,2 mg (0.22 mmol, 95.7 % coupling yield, 18.8 % total yield) linear peptide were obtained.
RP-HPLC: t_R = 22.9 min, gradient of 0 % to 50 % acetonitrile over 25 minutes in 0.1 % aqueous TFA at a flow rate of 1 ml/min. **HRMS (ESI):** m/z calcd for $C_{52}H_{70}N_{10}O_{11}S_2$: 1075.47 $[M+H]^+$; found = 1075.48, 538.24 $[M+2H]^{2+}$; found = 538.24.
136.7 mg (0,127 mmol) of crude linear OctF1PrgAlc were cyclized according to 2.2.9. 112,1 mg (0.104 mmol) of cyclized OctF1PrgAlc were obtained (82,0 % cyclization yield, 8.9 % overall yield).
RP-HPLC: t_R = 23.3 min, gradient of 0 % to 50 % acetonitrile over 25 minutes in 0.1 % aqueous TFA at a flow rate of 1 ml/min. **HRMS (ESI):** m/z calcd for $C_{52}H_{68}N_{10}O_{11}S_2$: 1073.46 $[M+H]^+$; found = 1073.46, 537.23 $[M+2H]^{2+}$; found = 537.23.

2.3.8. Synthesis of OctF3PrgAlc (1-A3)

For the synthesis of **1-A3** 0.4 g of 2-CTC resin (max. load: 1.46 mmol/g, 0.584 mmol) were preactivated according to 2.2.1. 0.556 g (1.45 mmol, 2.5 eq.) of Fmoc-Thr(tBu)-ol were coupled according to 2.2.3 b). After cleaving of the Fmoc protecting group the initial load was determined by UV/Vis spectroscopy to be 0.95 mmol/g (0.38 mmol, Yield of 65.1 %). Coupling of the Fmoc protected amino acids Thr, Cys, Thr, Lys, Trp, Phe, Cys and propargyl tyrosine was performed according to 2.2.4. After cleaving of the peptide from the resin according to 2.2.7, a crude yield of 361.1 mg (0.34 mmol, 89.5 % coupling yield, 58.2 % total yield) of linear peptide were obtained. All 361.1 mg (0.34 mmol) of crude linear OctF3PrgAlc were cyclized according to 2.2.8. 337.5 mg (0.31 mmol) of cyclized OctF3PrgAlc were obtained (91.1 % cyclization yield, 53.1 % overall yield).
RP-HPLC: t_R = 9.5 min. **HRMS (ESI):** m/z calcd for $C_{52}H_{68}N_{10}O_{11}S_2$: 1073.46 $[M+H]^+$; found = 1073.46, 537.23 $[M+2H]^{2+}$; found = 537.23.

2.4. Synthesis of TAMRA Conjugated Octreotide and Derivatives

2.4.1. Synthesis of OctF3PrgAc-TAMRA

73.1 mg (0.025 mmol) of the loaded resin from 2.3.2 were coupled with the linker according to 2.2.5 and with TAMRA-NHS according to 2.2.7. After cleaving with TFA according to 2.2.7, 40.7 mg (0.023 mmol) of linear peptide were obtained.
RP-HPLC: t_R = 13.9 min and 14.3 min. **HRMS (ESI):** m/z calcd for $C_{91}H_{114}N_{14}O_{21}S_2$: 1790.80 $[M+H]^+$; found = 1790.80.
After cyclization according to 2.2.8, 69.5 mg of crude cyclic peptide were obtained. The cyclic peptide was dissolved in 50 % aqueous ACN and purified by preparative RP-HPLC. 7.7 mg (4.3 µmol) of pure cyclic peptide were obtained.
RP-HPLC: t_R = 13.7 min and 14.1 min. **HRMS (ESI):** m/z calcd for $C_{91}H_{112}N_{14}O_{21}S_2$: 1787.78 $[M+H]^+$; found = 1787.78.

2.4.2. Synthesis of OctF1PrgAc-TAMRA

71 mg (0.026 mmol) of the loaded resin from 2.3.3 were coupled with the linker according to 2.2.5 and with TAMRA-NHS according to 2.2.7. After cleaving with TFA according to 2.2.7 38.7 mg (0.021 mmol) of linear peptide were obtained.
RP-HPLC: t_R = 14.2 min and 14.4 min. **HRMS (ESI):** m/z calcd for $C_{91}H_{114}N_{14}O_{21}S_2$: 1804.78 $[M+H]^+$; found = 1804.77.
After cyclization according to 2.2.8, 81.5 mg of crude cyclic peptide were obtained. The cyclic peptide was dissolved in 50 % aqueous ACN and purified by preparative RP-HPLC. 0.4 mg (0.22 µmol) of pure cyclic peptide were obtained.
RP-HPLC: t_R = 14.3 min and 14.5 min. **HRMS (ESI):** m/z calcd for $C_{91}H_{112}N_{14}O_{21}S_2$: 1801.76 $[M+H]^+$; found = 1801.77.

2.4.3. Synthesis of OctF3AllylAc-TAMRA

238 mg (0.103 mmol) of the loaded resin from 2.3.5 were coupled with the linker according to 2.2.5. Afterwards, TAMRA-NHS was coupled according to 2.2.7. The peptide was cleaved from the resin according to 2.2.7 to yield 137.4 mg (0.076 mmol) of linear peptide.
RP-HPLC: t_R = 14.4 min and 14.8 min. **HRMS (ESI):** m/z calcd for $C_{91}H_{116}N_{14}O_{21}S_2$: 1806.80 $[M+H]^+$; found = 1806.79.
Cyclization according to 2.2.8 yielded 148.5 mg of crude cyclic peptide. The cyclic peptide was dissolved in 50 % Aqueous ACN and purified by preparative RP-HPLC. 18.4 mg (0.01 mmol) of pure cyclic peptide were obtained.
RP-HPLC: t_R = 14.6 min and 15.0 min. **HRMS (ESI):** m/z calcd for $C_{91}H_{114}N_{14}O_{21}S_2$: 1803.78 $[M+H]^+$; found = 1803.78.

2.4.4. Synthesis of OctF1AllylAc-TAMRA

234 mg (0.01 mmol) of the loaded resin from 2.3.6 were coupled with the linker according to 2.2.5. Afterwards, TAMRA-NHS was coupled according to 2.2.7. The peptide was cleaved from the resin according to 2.2.7 to yield 132 mg (0.073 mmol) of linear peptide.
RP-HPLC: t_R = 14.3 min and 14.8 min. **HRMS (ESI):** m/z calcd for $C_{91}H_{116}N_{14}O_{21}S_2$: 1806.80 $[M+H]^+$; found = 1806.80.
Cyclization according to 2.2.8 yielded 285.4 mg of cyclic peptide. The cyclic peptide was dissolved in 50 % Aqueous ACN and purified by preparative RP-HPLC. After combining the fractions containing only the product 13.4 mg (7.4 µmol) of pure cyclic peptide were obtained.
RP-HPLC: t_R = 14.1 min and 14.6 min. **HRMS (ESI):** m/z calcd for $C_{91}H_{114}N_{14}O_{21}S_2$: 1803.78 $[M+H]^+$; found = 1803.78.

2.4.5. Synthesis of Fmoc-Trp-Lys-Thr-Cys-Thr-2-CTC for Conjugation with Fluorophore

0.456 g of 2-CTC resin (max. load: 1.46 mmol/g, 0.666 mmol) were preactivated according to 2.2.1 and coupled with Fmoc-Thr(tBu)-ol according to 2.2.3 (achieved initial load: 0.378 mmol/g, 0.172 mmol, Yield of 25.8 %). The amino acids cysteine, threonine, lysine and tryptophan were coupled according to 2.2.4 to yield 0.74 g of loaded resin, which were split for the following syntheses.

2.4.6. Synthesis of OctF3PrgAlc-TAMRA

0.246 g (0.057 mmol) of loaded resin from 2.4.5 were coupled to the amino acids propargyl tyrosine, cysteine and D-phenylalanine according to 2.2.4. The linker was coupled according to 2.2.5, and TAMRA-NHS was coupled according to 2.2.7. Cleaving of the peptide from the resin was performed according to 2.2.7 to yield 44.1 mg (0.025 mmol) of linear peptide.
RP-HPLC: t_R = 13.9 min and 14.3 min. **HRMS (ESI):** m/z calcd for $C_{91}H_{116}N_{14}O_{20}S_2$: 1789.80 $[M+H]^+$; found = 1789.80.
After cyclization according to 2.2.8, 103.4 mg of crude cyclic peptide were obtained. The cyclic peptide was dissolved in 50 % Aqueous ACN and purified by preparative RP-HPLC. 4.6 mg (2.6 µmol) of pure cyclic peptide were obtained.
RP-HPLC: t_R = 13.7 min and 14.1 min. **HRMS (ESI):** m/z calcd for $C_{91}H_{114}N_{14}O_{20}S_2$: 1787.78 $[M+H]^+$; found = 1787.78.

2.4.7. Synthesis of OctF1PrgAlc-TAMRA

0.495 g (0.115 mmol) of loaded resin from 2.4.5 were coupled with the amino acids phenylalanine and cysteine to yield 0.534 g of loaded resin. 0.39 g (0.084 mmol) of this resin were used for the following synthesis (2.4.8). The rest (0.144 g, 0.031 mmol) was coupled with propargyl tyrosine according to 2.2.4. The linker and TAMRA-NHS were coupled according to 2.2.5 and 2.2.7 respectively. The peptide was cleaved according to 2.2.7 to yield 48.2 mg (0.027 mmol) of linear peptide.
RP-HPLC: t_R = 13.91 min and 14.32 min. **HRMS (ESI):** m/z calcd for $C_{91}H_{116}N_{14}O_{20}S_2$: 1790.80 $[M+H]^+$; found = 1790.80.
After cyclization according to 2.2.8, 40.0 mg of crude cyclic peptide was obtained. The cyclic peptide was dissolved in 50 % Aqueous ACN and purified by preparative RP-HPLC. 7.4 mg (4.1 µmol) of pure cyclic peptide were obtained.
RP-HPLC: t_R = 13.86 min and 14.27 min. **HRMS (ESI):** m/z calcd for $C_{91}H_{114}N_{14}O_{20}S_2$: 1787.78 $[M+H]^+$; found = 1787.78.

2.4.8. Synthesis of Octreotide-TAMRA

0.39 g (0.084 mmol) of loaded resin from 2.4.7 were coupled to D-phenylalanine according to 2.2.4 yielding 0.413 g of loaded resin. 0.153 g (0.031 mmol) of this resin were coupled to the linker and TAMRA-NHS according to 2.2.5 and 2.2.6, respectively. The peptide was cleaved according to 2.2.7 to yield 49.3 mg (0.028 mmol) of linear peptide.
RP-HPLC: t_R = 13.6 min and 14.0 min. **HRMS (ESI):** m/z calcd for $C_{88}H_{114}N_{14}O_{19}S_2$: 1736.79 $[M+H]^+$; found = 1736.79.
After cyclization according to 2.2.8, 47.2 mg of crude cyclic peptide were obtained. The cyclic peptide was dissolved in 50 % Aqueous ACN and purified by preparative RP-HPLC. After combining the fractions containing only the product 4.5 mg (2.5 µmol) of pure cyclic peptide were obtained.
RP-HPLC: t_R = 13.4 min and 13.8 min. **HRMS (ESI):** m/z calcd for $C_{88}H_{112}N_{14}O_{19}S_2$: 1733.77 $[M+H]^+$; found = 1733.77.

3. NMR Methods

Methanol-d_4 (>99.8% deuteration) was purchased from Deutero and Sigma Aldrich, the catalyst [1,4-bis-(diphenylphosphino)-butan]-(1,5-cyclooctadien)-rhodium(I)-tetrafluoroborat ([Rh(dppb)(COD)]BF4) and D_2O (99.9 %) from Sigma-Aldrich. NMR experiments were performed in a 11.7 T OXFORD 500 MHz magnet equipped with a Bruker AVANCE III HD spectrometer.
The parahydrogen enrichment was performed with a parahydrogen generator from Advanced Research Systems Inc. comprising a DE204A cryostat and an ARS-4HW compressor. The cryostat is cooled to 30 K. >95 % para-enriched hydrogen was delivered into the NMR sample tube placed inside the magnet at elevated pressures and room temperature. A custom-made setup was used for bubbling directly in the tube.[8] A standard 5 mm screw-cap NMR sample tube (from Rototec Spintec, 5mm 528-TR-7) was used, closed by a cap-adapter with an in- and outlet for the gases. A thin glass capillary was attached to the inlet and immersed in the sample. The closed system was pressurized with helium, and gas flow was maintained due to a small pressure difference between the inlet and outlet at 7 bar. The gas was supplied through magnetic valves controlled by the pulse program of the NMR spectrometer, allowing not only to switch between helium and parahydrogen, but also to apply vacuum to the input/output to avoid diffusion mixing of gases in the supply tube. For maximal signal enhancement, we took care that the polarized product was formed rapidly within the time window given by T_1-relaxation. The bubbling process was synchronized with the NMR pulses and was stopped 2 s before the detection of NMR spectra.

3.1. PHIP Experiments

For the PHIP experiments stem solutions of 5 mg/ml peptide (150 µl used per sample) and 3 mg/ml catalyst were prepared. They were mixed with deuterated methanol to a total volume of 690 µl to give a final 1 mM peptide concentration and three different catalyst concentrations: 0.45 mM (75 µl of stem solution), 0.9 mM (150 µl) and 1.8 mM (300 µl). The NMR tube was connected to an adapter holding a glass capillary, inserted into the magnet, filled with helium under pressure and bubbled with para-enriched hydrogen gas for 15-30 s at 7 bar. After waiting for 2 seconds a standard PHIP sequence was applied by irradiation of a 45° pulse followed by the acquisition. All PHIP-spectra were recorded as "single-shot experiments", performing one scan. Full relaxation of the polarized protons in the products was observed at least 2 minutes after the hydrogenation.
For the experiments in mixtures of methanol and water with up to 50 % of D_2O, samples were prepared as before, but D_2O was added instead of methanol to obtain the final sample volume and concentrations. For amounts higher than 50 % D_2O, the peptide stem solution had to be prepared in D_2O. This way the only methanol added originated from the catalyst stem solution. Similarly, samples were prepared in a 50 % ethanol mixture from a 2.5 mg/ml catalyst stem solution in ethanol-d6 (360 µl) and the peptide dissolved in D_2O. To prevent the formation of foam, 10 µl of a 10 % TMPB solution in hexamethyldisiloxane were added as a thin layer onto the sample.

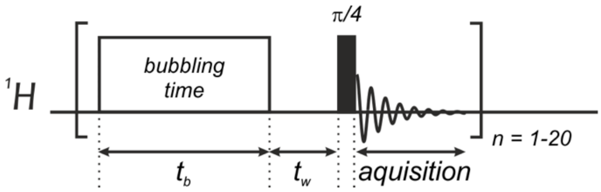

Figure S-6: Pulse sequence for PHIP experiments. Bubbling time t_b = 15 to 30 seconds, wait time, t_w = 2 seconds.

3.1.1. Enhancement Factors

Signal Enhancements (SE) were calculated from the absolute integrals (*AInt*) of the respective signal using MestreLab Research MestReNova 14.2. Since the sum of the integrals of the antiphase signals is equal to zero, *AInt(PHIP)* was determined as the sum of the moduli of the emission and absorption areas of the respective antiphase signal. All absolute integrals were divided by the NMR receiver gain (*rg*) and the number of scans (*ns*). In those cases where the peptide concentration deviated from 1 mM, the integral was also normalized to this value by dividing the integral by the peptide concentration (c_p) in mmol/l. The signal enhancement was calculated as quotient of the normalized integral *NInt(PHIP)* for the polarized signal and the normalized integral *NInt(therm.)* for the signal in thermal equilibrium following equation:

$$SE \approx \frac{NInt(PHIP)}{NInt(therm.)} = \frac{\frac{AInt(PHIP)}{rg \times ns\ (\times c_p)}}{\frac{AInt(therm.)}{rg \times ns}}$$

Due to the strong broadening and partial shifting of the allyl signals in relaxed spectra after the reaction (Figure S-7), thermal signals were weak, leading to lower values for *Int(therm.)* and an overestimation of the signal enhancement. The further reaction of the allyl moiety to a propyl moiety also could not be ruled out completely, especially for the marker molecule **A** (see chapter 3.4 in SI). Therefore, the corresponding signals of the allyl variants **8** and **9**, measured at the same concentration of 1 mM without undergoing the reaction, were used as external standard for *Int(therm.)*. These integrals would correspond to the thermal integrals observed for the reaction products after hydrogenation and relaxation of the hyperpolarization, if a complete reaction of the propargyl moiety of the PHIP marker to the allyl moiety and no further reaction to the propyl moiety is assumed.
The broadening and signal shift is caused by the binding of the catalyst to some of the peptide molecules either as intermediate product-catalyst complexes or by interactions with aromatic moieties of the peptide.[9,10]
Table S-1 and Table S-2 summarize the signal enhancements in the three spectral regions, 5.23, 5.35 and 6.02 ppm at different catalyst concentrations.

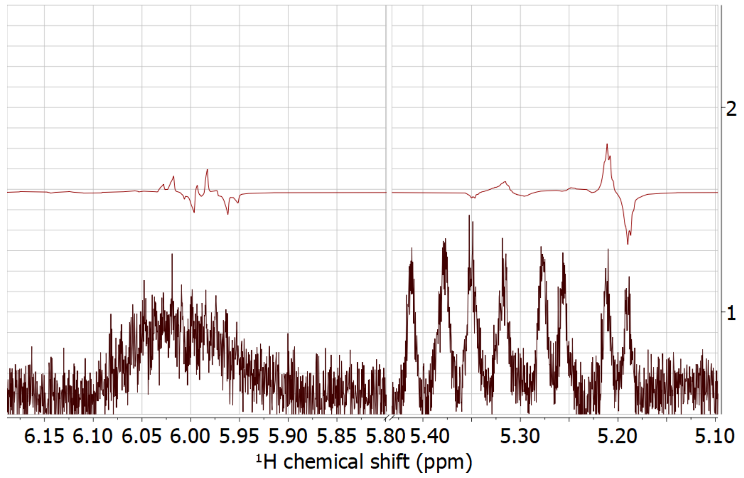

Figure S-7: Signals corresponding to the allyl moiety in the PHIP spectrum (top, rg = 3.9, ns = 1) and relaxed thermal spectrum (bottom, rg = 287, ns = 72) of **2-A1**. In the relaxed spectrum the signals appear broadened and doubled, indicating the presence of a free product and a catalyst bound intermediate.

Table S-1: Signal enhancement factors for different octreotide derivates and PHIP marker Fmoc-propargyltyrosine at different catalyst concentrations in MeOD-d_4 with respective reaction times for the three different hyperpolarized signals around 5.23 ppm (H^B), 5.35 ppm (H^A) and 6.02 ppm (H^C). Averages of three measuremens for the peptides and two for the marker **A**.

Sample	Catalyst Conc.	H^C 6.02 ppm	H^A 5.35 ppm	H^B 5.23 ppm
F1PrgAlc (**1-A1**)	High (1.8 mM)	447 ±136, 25 s	732 ±89, 25 s	1374 ±506, 25 s
	Medium (0.9 mM)	193 ±71, 25 s	253 ±37, 25 s	488 ±191, 20 s
	Low (0.45 mM)	110 ±17, 25 s	90 ±8, 25 s	324 ±51, 25 s
F1PrgAc (**2-A1**)	High (1.8 mM)	584 ±93, 20 s	1049 ±233, 20 s	1251 ±237, 25 s
	Medium (0.9 mM)	383 ±95, 23 s	349 ±114, 23 s	890 ±156, 23 s
	Low (0.45 mM)	120 ±19, 25 s	67 ±6, 25 s	364 ±58, 25 s
F3PrgAlc (**1-A3**)	High (1.8 mM)	306 ±58, 25 s	43 ±5, 25 s	242 ±40, 25 s
	Medium (0.9 mM)	71 ±13, 25 s	10 ±1, 25 s	66 ±19, 25 s
	Low (0.45 mM)	25 ±13, 25 s	15 ±15, 30 s	37 ±12, 25 s
F3PrgAc (**2-A3**)	High (1.8 mM)	560 ±105, 21 s	98 ±10, 21 s	731 ±114, 21 s
	Medium (0.9 mM)	184 ±35, 25 s	50 ±12, 25 s	309 ±38, 25 s
	Low (0.45 mM)	124 ±23, 35 s	26 ±3, 40 s	184 ±21, 35 s
Fmoc-Tyr(Prg)-OH (**A**)	High (1.8 mM)	1128 ±155, 25 s	318 ±83, 25 s	1881 ±745, 25 s
	Medium (0.9 mM)	1471 ±166, 25 s	350 ±45, 25 s	2875 ±299, 25 s
	Low (0.45 mM)	1787 ±172, 20 s	397 ±80, 25 s	3438 ±323, 25 s

Table S-2: Signal enhancement factors for **2-A1** at different catalyst concentrations in D_2O/MeOD-d_4 mixtures with respective reaction times in seconds for the three different hyperpolarized signals around 5.25 ppm (H^A), 5.35 ppm (H^B) and 6.0 ppm (H^C). Due to broadening and overlap the H^A signals could not be clearly distinguished from those of H^B in 50 % D_2O, thus only enhancement values for clearly distinguishable H^B signals are given.

Sample	Catalyst Conc.	H^C 6.02 ppm	H^A 5.35 ppm	H^B 5.23 ppm
F1PrgAlc (**1-A1**) 50 % EtOD-d_6	High (1.8 mM)	163 ±26, 20 s	60 ±5, 25 s	466 ±74, 20 s
F1PrgAc (**2-A1**)	High (1.8 mM)	331 ±60, 15 s	-	896 ±152, 15 s
50 % D_2O/MeOD-d_4	Medium (0.9 mM)	310 ±49, 25 s	-	822 ±130, 25 s
	Low (0.45 mM)	162 ±25, 20 s	-	449 ±71, 20 s
64 % D_2O/MeOD-d_4	Low (0.45 mM)	281 ±44, 25 s	102 ±9, 25 s	815 ±129, 25 s

3.2. Time Savings

In theory a SE of 1000 leads to a time saving factor of about 1 000 000. A SE of 2000 would correspond to a factor of 4 000 000. As the hydrogenation reaction before the measurement also takes time (typically 25 seconds), it needs to be considered to determine a practical time saving factor. This can be done based on the Signal to Noise Ratio (SNR).
A usual PHIP experiment including the reaction time takes about 30 seconds. With a SE of 1000 the (SNR) is up to 340. A normal ^{1}H spectrum with 32 scans takes about 256 seconds (3 s acquisition and 5 s d1, 8 s per scan). In thermal equilibrium at room temperature the SNR is about 12.
For twice the SNR the number of scans needs to be quadrupled. It would take about 25 686 scans at thermal equilibrium to achieve the same SNR of 340 of the PHIP experiment, which would take about 205 488 s. The PHIP experiment is 6 850 times faster.
For the SE of 2 000 the SNR was 1 300. It would take about 375 518 scans or 3 004 444 s at thermal equilibrium, leading to a time advantage for the PHIP experiment of about 100 000.

3.3. Kinetic Measurements

PHIP kinetics were measured by repeating standard PHIP measurements, described in 3.1. with 5 s bubbling time right after each other. It allows to track the maximum speed of reaction and its decline with increased total reaction time.
A second method used a single 15 second bubbling period followed by repeated 3 second delays and a short 5° pulse followed by 3 seconds of acquisition. This way we can track the polarization while hardly depolarizing the sample with the pulse (cos(5°) = 99.6 % of remaining polarization).

3.4. 2D-TOCSY Experiments

For the Total Correlation Spectroscopy (TOCSY) we employed a standard dipsy2phpr experiment (Figure S-8). The dimension T_{d1} was set to 1024 and 16 scans were employed for each slice. The FnMode was chosen as TPPI. The presaturation was done for d1=4 seconds at 3.9811e-5 W at the frequency of the solvent signal at 4.84 ppm. The delay d0 is incremented by 200 µs for each slice in T_{d1}. The DIPSI-2 block was D9=80 ms long, with the delays d20=2 ms before and d21=3 ms afterwards.
The concentration of the analytes was 5 mg/ml. The measurements were performed once before the reaction and two times after hydrogenation.

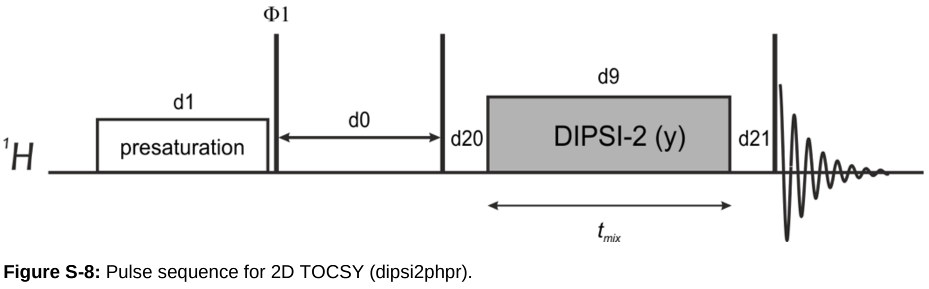

Figure S-8: Pulse sequence for 2D TOCSY (dipsi2phpr).

Figure S-9 shows the 2D-TOCSY spectra of Fmoc-Tyr(Prg)-OH (**A**) before, right after and long after starting the hydrogenation reaction. It can be seen that for the spectrum right after the reaction new signals for H^A, H^B and H^C emerge between 5.2 and 6.0 ppm which form a coupling pattern as expected for the allyl group. The reaction continues with the dissolved hydrogen leading to a new coupling pattern between 1.0 and 3.8 ppm indicating the formation of a

propyl group. Figure S-10 shows the aromatic region of the spectra. Here the shift of the tyrosine signals with the ongoing reaction is apparent. Both of these observations indicate a complete reaction from the alkynyl via the alkenyl to the alkyl group.

For the peptide **2-A3** only the first reaction step to the alkenyl is apparent in the spectra shown in Figure S-11 and Figure S-12. This indicates that the second reaction step is either much slower or hindered in the peptide.

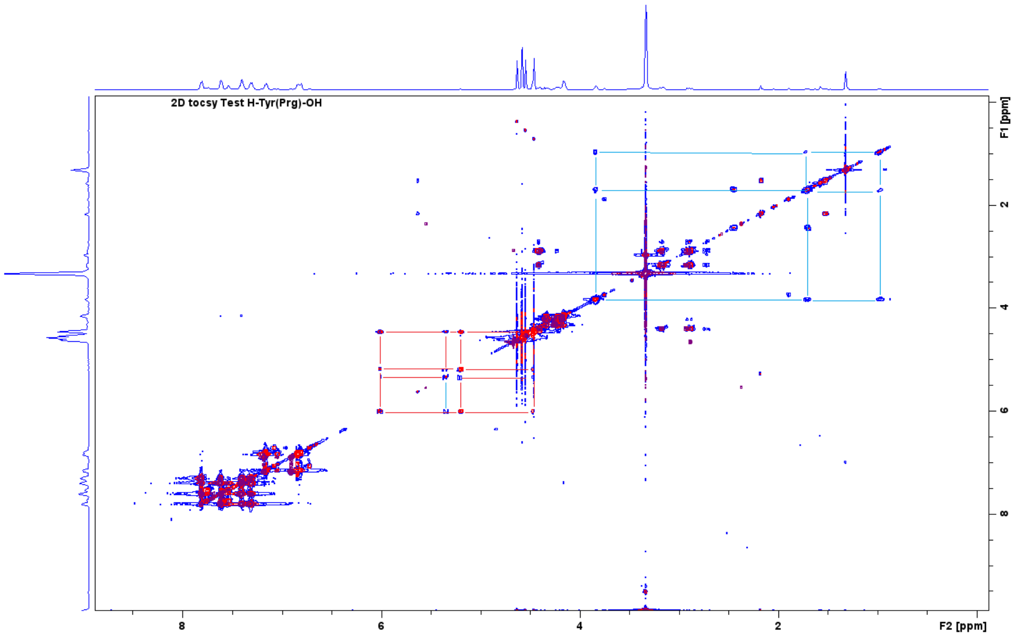

Figure S-9: 2D-TOCSY of Fmoc-Tyr(Prg)-OH before (purple), shortly after hydrogenation (red) and later (blue). The coupling of signals of the allyl group is marked with the red squares and diminishes with time while the signals of a propyl group emerge; the respective coupling is marked with blue squares.

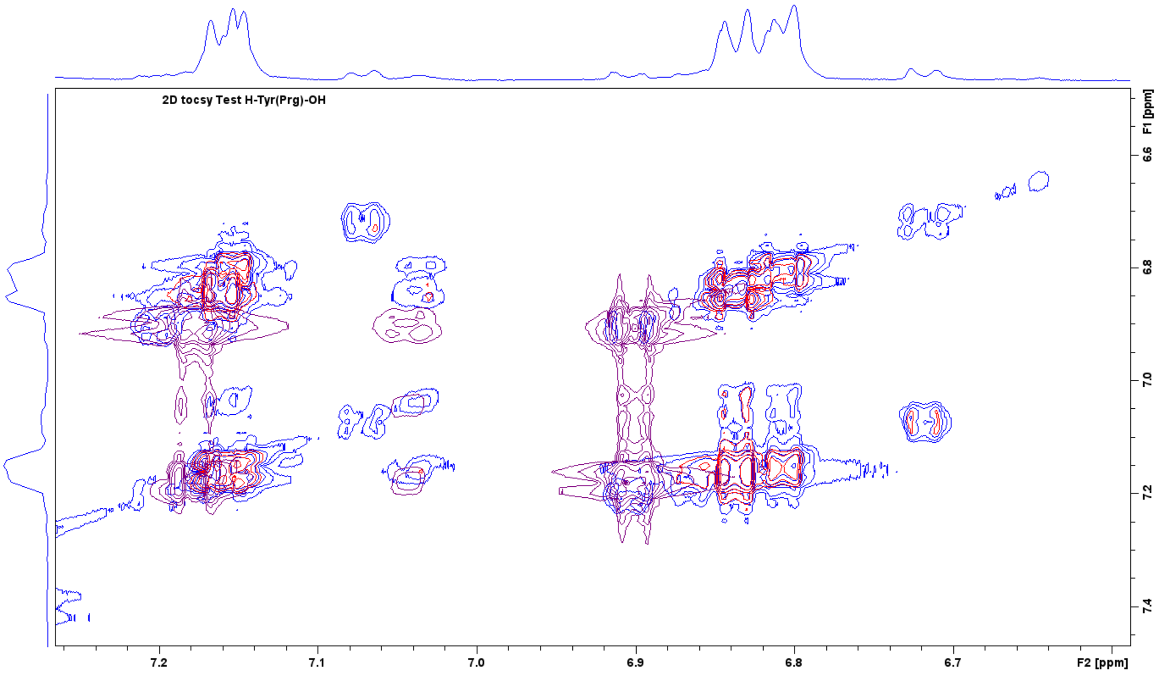

Figure S-10: Aromatic region of the 2D -TOCSY spectrum of Fmoc-Tyr(Prg)-OH before (purple), after (red) and long after (blue) hydrogenation. One of the signals of tyrosine shifts visibly from about 6.90 via 6.85 to about 6.81 ppm. A weaker shift of the second tyrosine signal at about 7.25 ppm is also apparent.

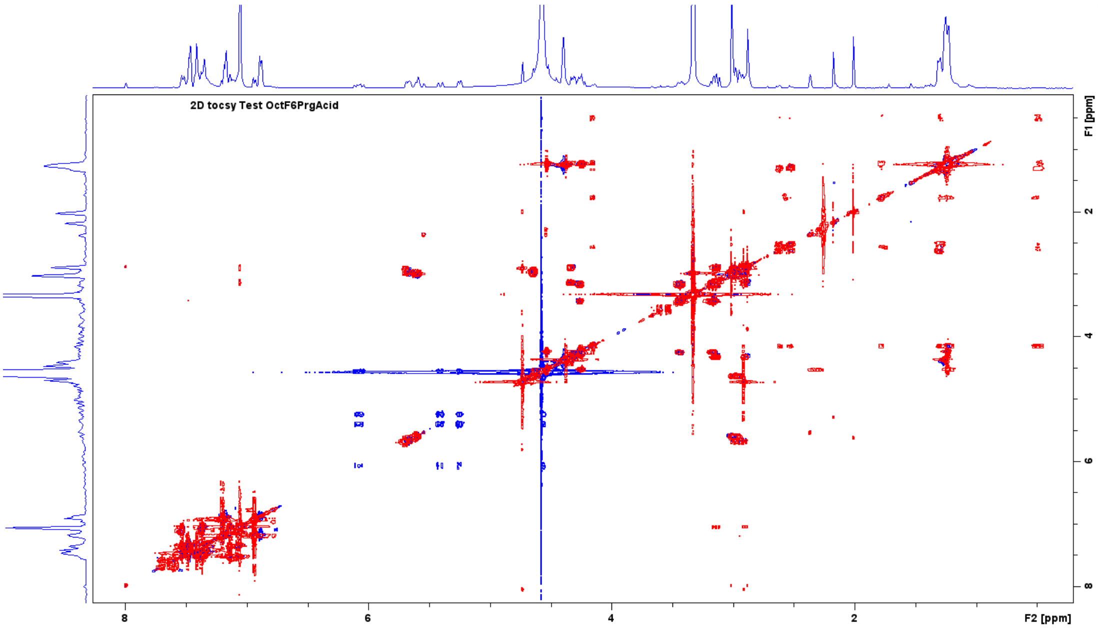

Figure S-11: 2D-TOCSY of OctF3PrgAc (**2-A3**) before (red) and after hydrogenation (blue). The coupling pattern of the allyl group signals after hydrogenation is well apparent. No new coupling pattern group in the lower half of the spectrum corresponding to an alkyl emerges.

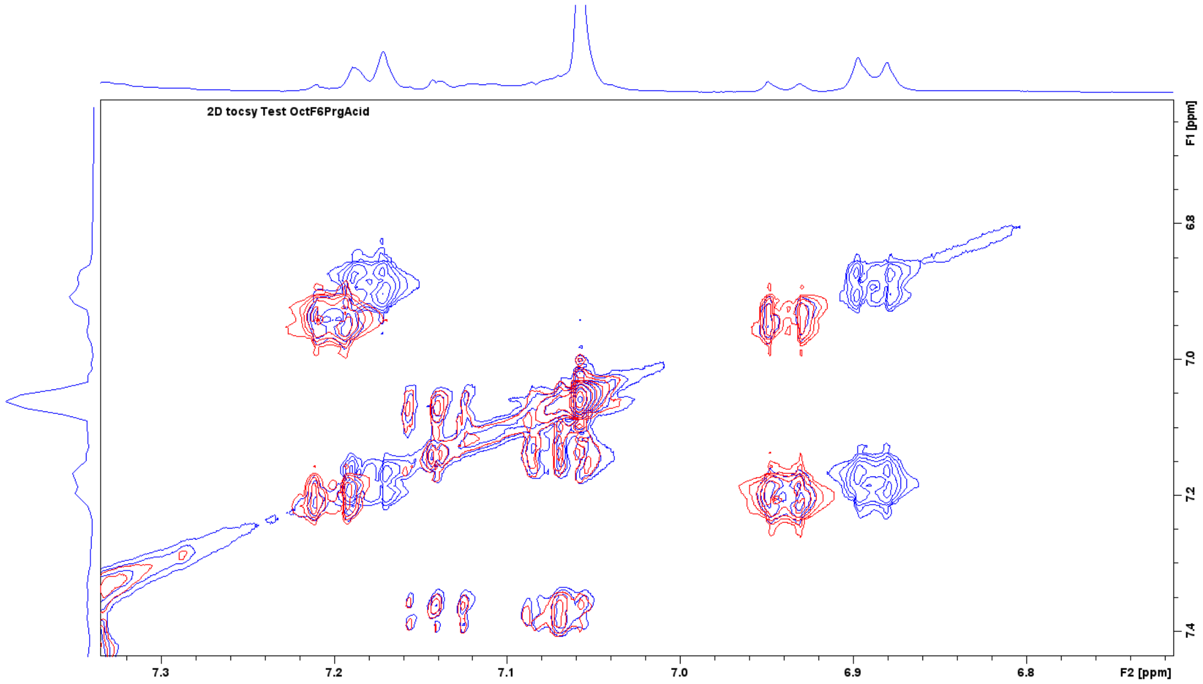

Figure S-12: Aromatic region of the 2D-TOCSY spectrum of OctF3PrgAc (**2-A3**) before (red) and after (blue) the hydrogenation reaction. One of the signals of tyrosine shifts visibly from about 6.94 to 6.89 ppm in only one step. A weaker shift of the second tyrosine signal at about 7.20 ppm is also apparent.

3.5. ^1H T_1 Measurements

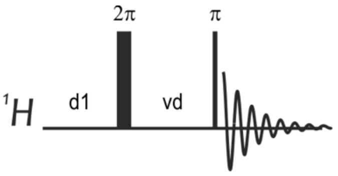

Figure S-13: Pulse sequence for the inversion recovery experiment.

T_1 was determined for the allyl signals of the variants **2-B1** and **2-B3** as the hydrogenation products by inversion recovery experiments (Figure S-13). The delay times vd of 0.01, 0.1, 0.25, 0.5, 1, 2, 3, 4, 5, 7.5 and 10 seconds were used. Spectra were measured with 8 scans and a d1 of 10 seconds for **2-B1** and 20 seconds for **2-B2**. A regular ^1H spectrum was measured to determine the maximum integral I_0 of the signals. The logarithm of the difference *ln*(I_0-*I(t))* was plotted against the delay time t. T_1 is determined by the inverse of the slope of a fitted linear function according to:

$$\ln\left(I_0 - I(t)\right) = -\frac{t}{T_1} + \ln\left(2I_0\right)$$

Table S-3 shows the resulting fit parameters and T_1 times for each investigated proton signal. The corresponding data and linear fits are shown in Figures S-14 and S-15.

Table S-3: Fit parameters for the inversion recovery data and results for ^1H T_1.

Proton signal, chemical shift, ppm	Fit parameters OctF1AllAc	T_1 time for OctF1AllAc (**2-B1**), s	Fit parameters OctF3AllAc	T_1 time for OctF3AllAc (**2-B3**), s
H^A, 5.35	-0.3348 s^{-1}*t+10.105	2.99	-0.2082 s^{-1}*t+10.111	4.80
	R^2 = 0.9805		R^2 = 0.9974	
H^B, 5.25	-0.3187 s^{-1}*t+10.143	3.14	-0.2777 $^{-1}$*t+10.207	3.60
	R^2 = 0.9992		R^2 = 0.993	
H^C, 6.00	-0.3920 s^{-1}*t+10.182	2.55	-0.2787 $^{-1}$*t+10.150	3.59
	R^2 = 0.9965		R^2 = 0.9936	

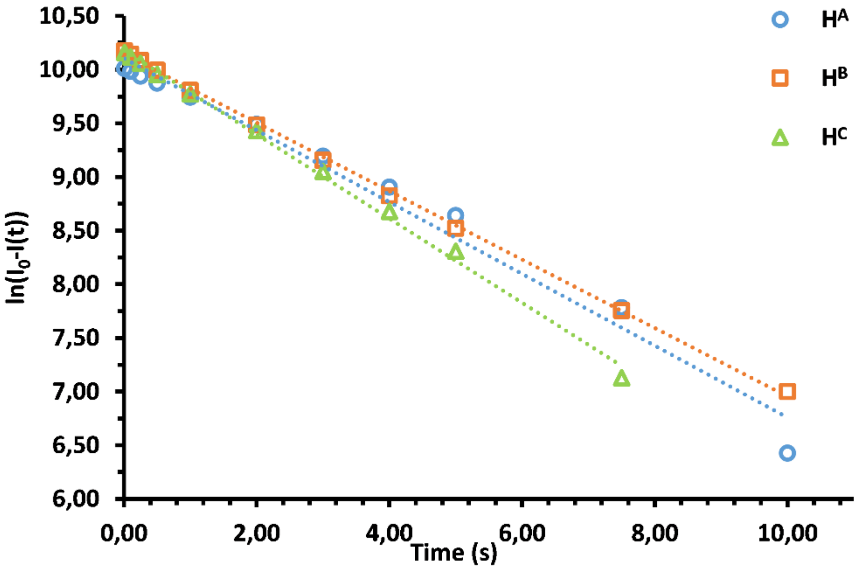

Figure S-14: Plot of ln(I_0-I(t)) against recovery time for the inversion recovery experiment of OctF1AllAc (**2-B1**). The point at t=10 s for H^C was omitted as I(t) was equal to I_0.

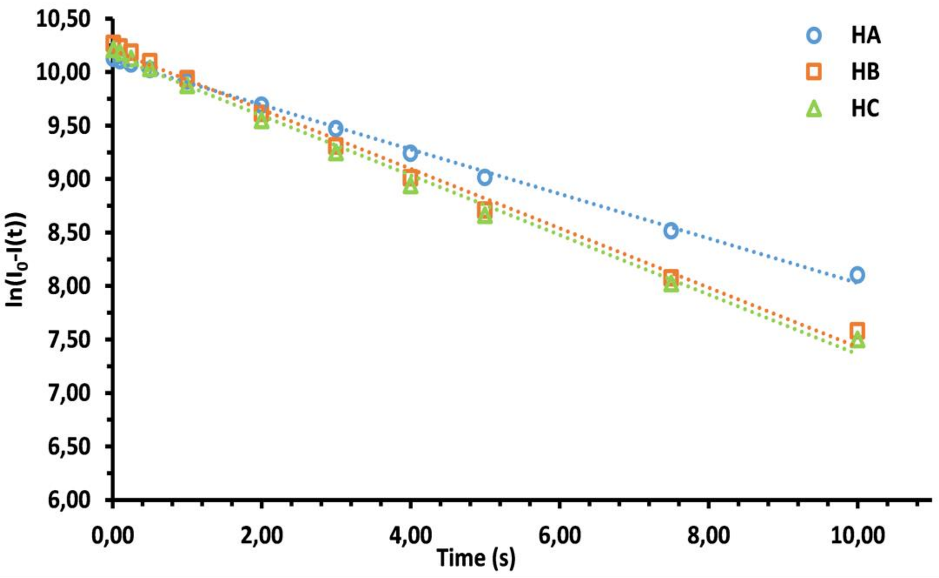

Figure S-15: Plot of ln(I_0-I(t)) against recovery time for the inversion recovery experiment of OctF3AllAc (**2-B3**).

3.6. ^{13}C T_1-Measurements

Analogous to the T_1 measurements for ^{1}H the T_1 times of ^{13}C were determined by inversion recovery experiments. The measurements were done for the unprotected amino acid H-L-Tyrosine(OAllyl)-OH (Figure S-16) in a 30 to 33 % aqueous solution of ammonia. Each spectrum was measured in 256 scans and without proton decoupling. The delay times vd were 0.2, 0.6, 1, 3, 6, 10, 30 and 60 seconds with a d1 time of 60 seconds. Measurements with delays of 120 and 300 seconds showed no further increase in the signal intensities, however, due to the noise level the integrals of those measurements were sometimes slightly smaller than those acquired with 60 seconds delay (Figure S-17). For the linearization of the data the integral value of a simple one-pulse measurement with a d1 of 60 seconds was used for I_0.
The plots of the linearized datasets deviate from the linear behavior after the first six to seven data points (Figure S-18 and Figure S-19). This could be caused by the low signal to noise ratio that leads to significant differences in the obtained integral values even after full recovery of the respective signals. Table S-4 shows the fit parameters and the resulting T_1 times for the fits of the first six to seven data points for each dataset. Besides the allyl carbons A, B and C also the quaternary carbons D and E were investigated. The latter two showed the longest T_1 times.

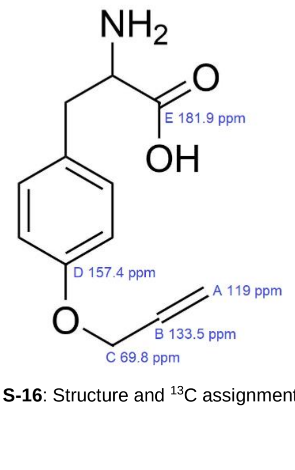

Figure S-16: Structure and ^{13}C assignments of H-L-tyrosine(allyl)-OH.

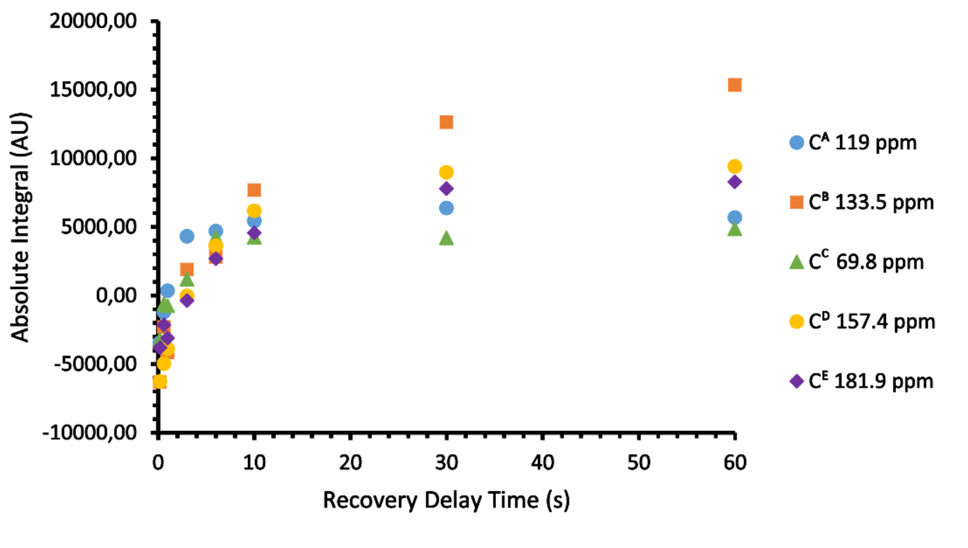

Figure S-17: Absolute integrals of the respective carbon signals against the recovery time for the inversion-recovery experiment on H-L-tyrosine(allyl)-OH.

Table S-4: Fit parameters for the inversion recovery data and results for ^{13}C T_1.

Carbon signal, chemical shift, ppm	Fit parameters	T_1 time, s
C^A, 119.0	-0.1382 s^{-1}*t+8.6899	7.24 ±0.94
	R^2 = 0.9228	
C^B, 133.5	-0.0904 s^{-1}*t+9.7961	11.07 ±0.59
	R^2 = 0.9859	
C^C, 69.8	-0.2192 s^{-1}*t+8.9029	4.56 ±0.81
	R^2 = 0.8874	
C^D, 157.4	-0.0867 s^{-1}*t+9.5266	11.53 ±1.22
	R^2 = 0.9474	
C^E, 181.9	-0.0546 s^{-1}*t+9.4556	18.33 ±1.93
	R^2 = 0.9474	

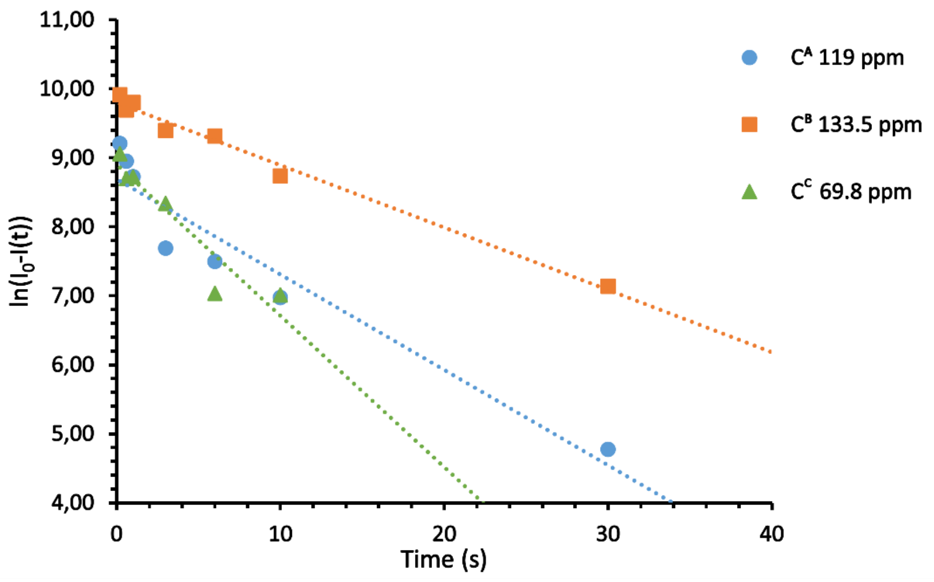

Figure S-18: Plot of the linearized data for the signals of the allyl residue at 119.0 ppm, 133.5 ppm and 69.8 ppm. Solid points are fitted with a linear equation, hollow points deviate from that linear behaviour.

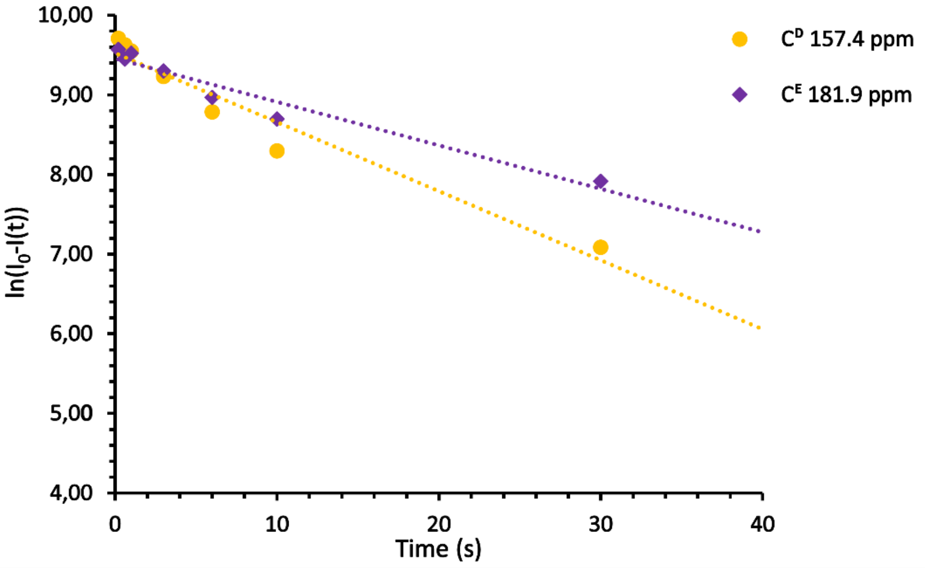

Figure S-19: Plot of the linearized data for the signals of the quaternary carbons at 157.4 ppm and 181.9 ppm. Solid points are fitted with a linear equation, hollow points deviate from that linear behaviour.

4. Cell Binding Assay

The cell binding assay was performed on A549[11] and HEK293[12] cells, which were tested for expression of somatostatin receptors of the type SSTR2 (Figure S-20). The cell lines were purchased from the German Collection of Microorganisms and Cell Cultures (DSMZ), catalogue numbers ACC 107 (A549) and ACC 308 (HEK293).

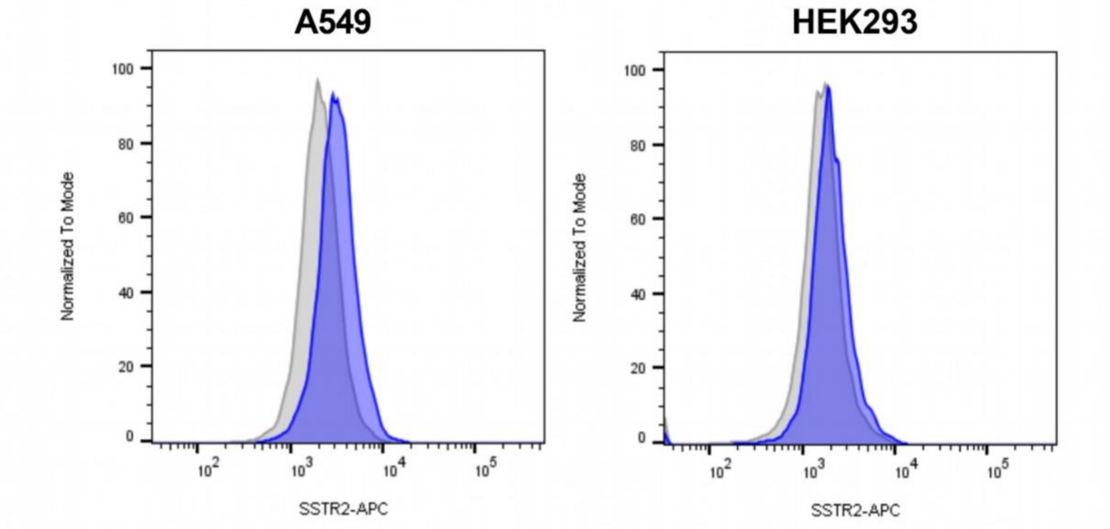

Figure S-20: Verification of SSTR2 expression for A549 and HEK293 cells by flow cytometry. An allophycocyanin (APC)-conjugated anti-SSTR2 antibody was used for intracellular staining (blue) compared to unstained cells (grey).

A549 cells (DSMZ ACC 107) and HEK293 cells (DSMZ ACC 308) were cultured in complete growth medium consisting of Dulbecco's MEM with 10 % fetal bovine serum (FBS) and 1x penicillin/streptomycin. The cells were sub-cultured every 3-4 days and maintained in a humified atmosphere at 37 °C and 5 % CO_2. To perform cell binding experiments, A549 cells were trypsinized and washed with ice-cold PBS containing 2 % FBS (PBS-F). In a 96-well U-bottom plate, 100 000 cells/well were seeded for staining. Cells were stained with the respective TAMRA-conjugated octreotide variant in PBS-F and incubated on ice for 30 min. After incubation, the cells were washed three times with ice-cold PBS-F. Using a CytoFlexS flow cytometer, PE-fluorescence was measured. Measurements were carried out in biological duplicates and graphs were plotted using GraphPad Prism 8.0.1 (Figure S-21and Figure S-22).

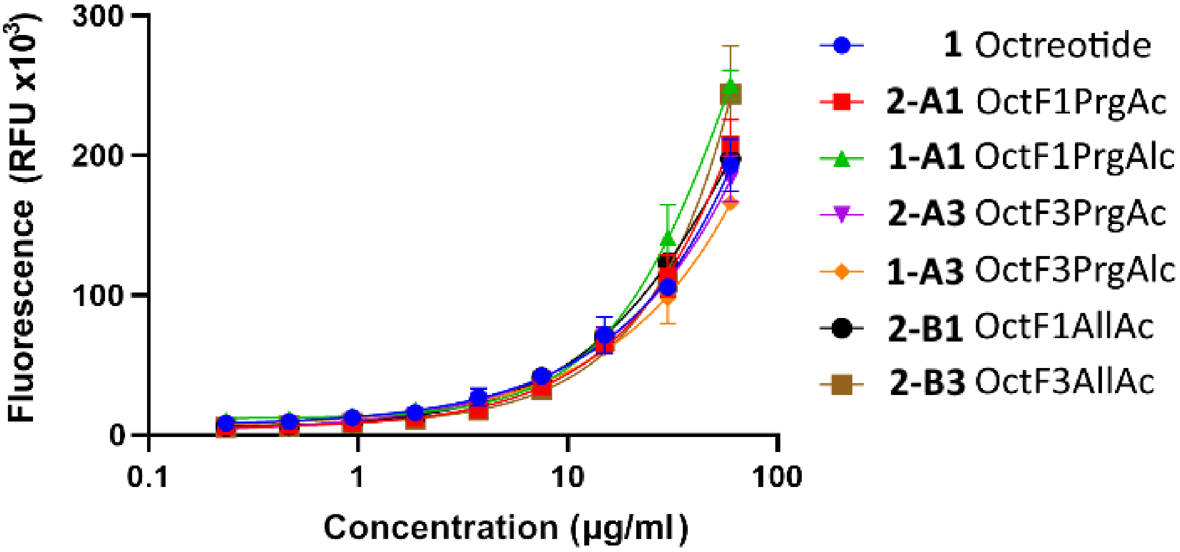

Figure S-21: Fluorescence of the TAMRA conjugated octreotide variants bound to the A549 cells against their concentration during incubation.

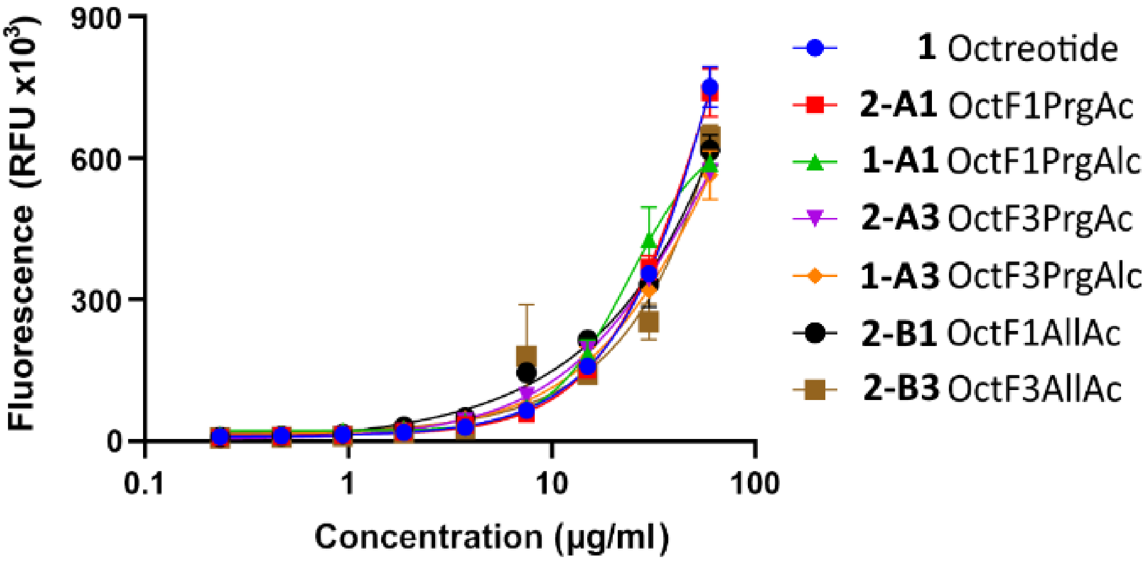

Figure S-22: Fluorescence of the TAMRA conjugated octreotide variants bound to the HEK293 cells against their concentration during incubation.

When the cells are incubated with neat TAMRA, the show about half the fluorescence compared to when treated with the fluorescence marked octreotide. This confirms that the binding affinity of neat TAMRA to the A549 cells as well as to HEK293 cells is weaker than the binding affinity of octreotide (Figures S-23 and S-24), which indicates a specific binding of peptides to the SSTRs.

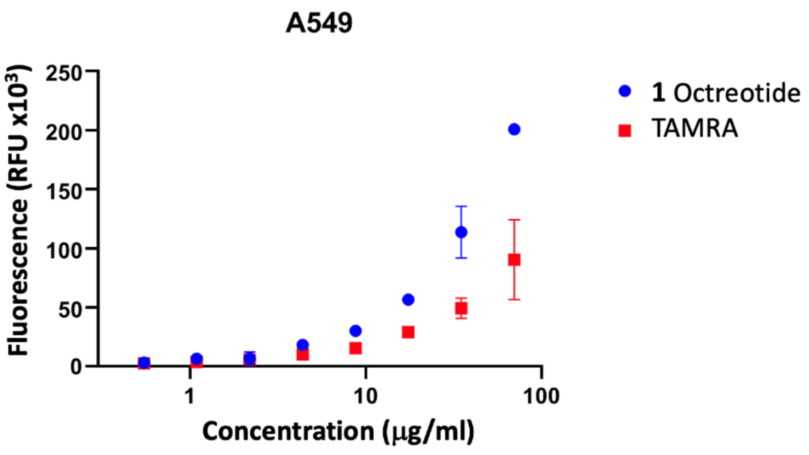

Figure S-23: Fluorescence of the TAMRA versus octreotide **1** bound to the A549 cells against their concentration during incubation.

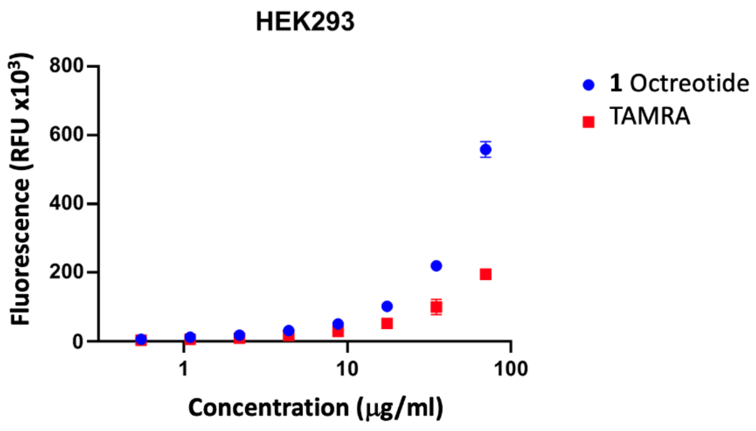

Figure S-24: Fluorescence of the TAMRA versus octreotide **1** bound to the HEK293 cells against their concentration during incubation.

5. Stability of the Disulfide Bond after Hydrogenation

The stability of the disulfide bond was tested by comparing the HPLC chromatograms of the folded peptide OctF1PrgAc before and after hydrogenation with those of the folded reaction product OctF1AllAc before and after hydrogenation as well as the linear precursor of OctF1AllAc without disulfide bond. The gradient used was 20 % to 80 % of 0.1 % TFA in acetonitrile over 20 minutes at 1 ml/minute.

The retention time of the linear OctF1AllAc is 11.027 minutes. The folded OctF1AllAc has a retention time of 11.560 minutes before and 11.567 minutes after hydrogenation basically remaining unchanged. This indicates no unfolding of the peptide has occurred during the reaction (Figure S-25).

The retention time of OctF1PrgAc before hydrogenation is 10.991 minutes and 11.635 minutes after hydrogenation, roughly matching the retention time of the folded reaction product OctF1AllAc. This indicates that the hydrogenation reaction occurred as expected and the disulfide bond remained stable (Figure S-26).

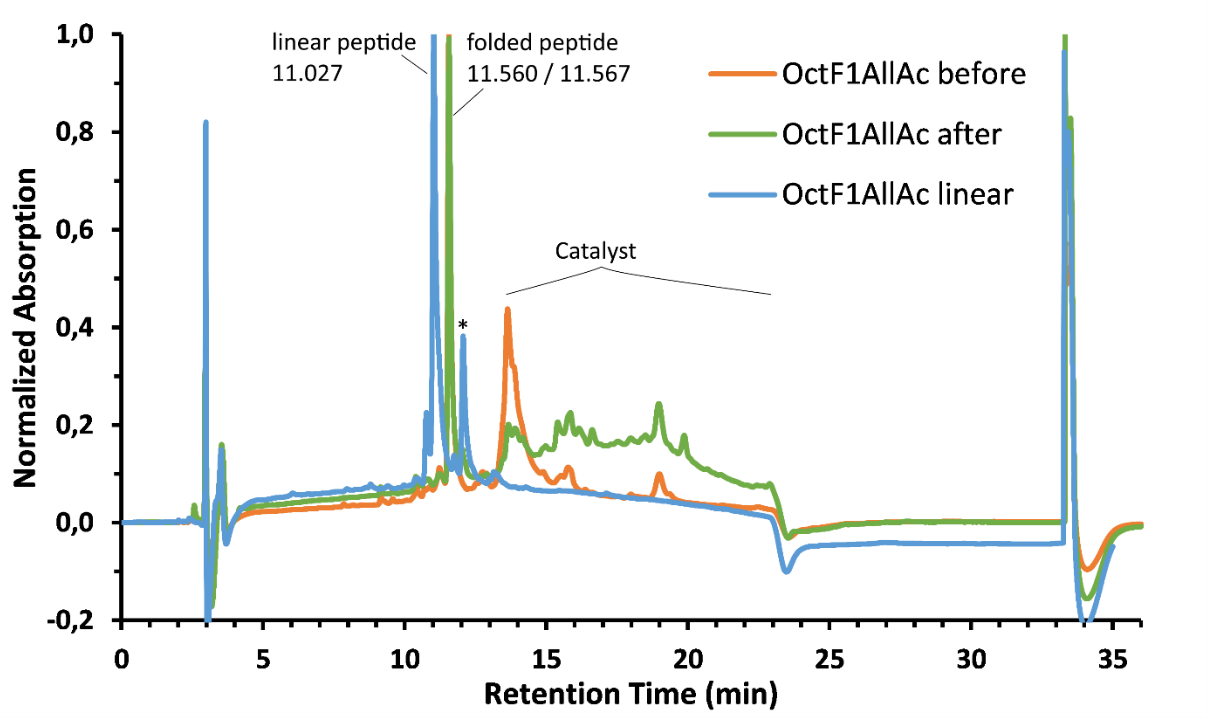

Figure S-25: RP-HPLC chromatogram at 214 nm of cyclic OctF1AllAc before and after hydrogenation and linear OctF1AllAc. Gradient of 20 % to 80 % acetonitrile over 20 minutes in 0.1 % aqueous TFA at 1 ml/min and subsequent purging at 95 % acetonitrile. The peaks after 13 min correspond to the catalyst in unreacted and reacted form. Peaks with a * correspond to impurities in the sample.

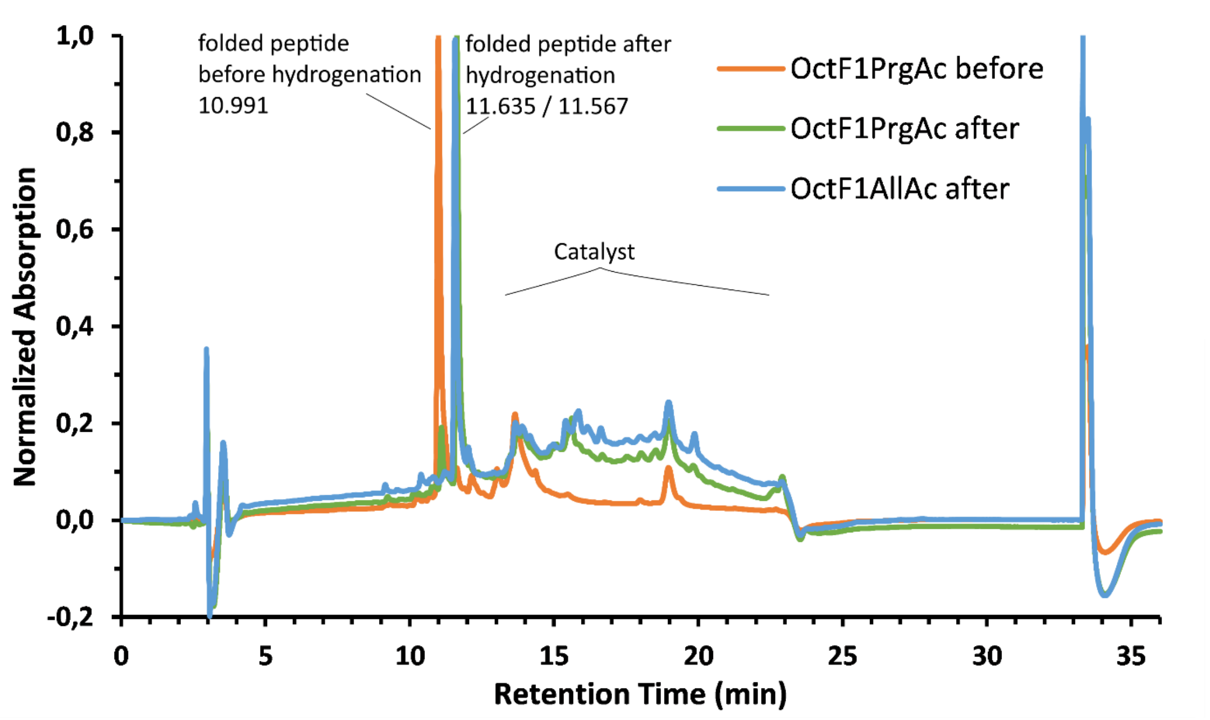

Figure S-26: RP-HPLC chromatogram at 214 nm of cyclic OctF1AllAc before and after hydrogenation and linear OctF1AllAc. Gradient of 20 % to 80 % acetonitrile over 20 minutes in 0.1 % aqueous TFA at 1 ml/min and subsequent purging at 95 % acetonitrile. The peaks after 13 min correspond to the catalyst in unreacted and reacted form.

6. RP-HPLC Chromatograms

The RP-HPLC chromatograms in this section show the obtained amino alcohol and octreotide samples after the synthesis. The samples with the TAMRA fluorescence marker and spacer were purified by preparative RP-HPLC and the fractions shown here were united. Due to the two isomers of TAMRA used those samples show up to two main peaks. Only those fractions containing either one or both of the product peaks were united.

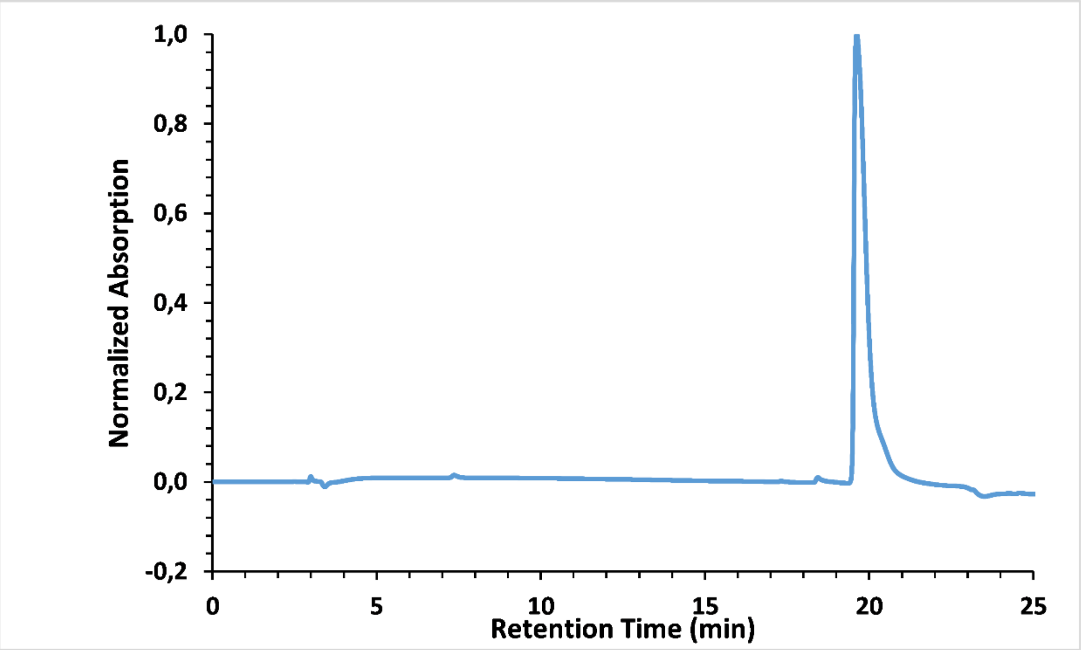

Figure S-27: RP-HPLC chromatogram at 214 nm of Fmoc-Thr(tBu)-ol after Flash-chromatography. Gradient of 20 % to 80 % acetonitrile over 20 minutes in 0.1 % aqueous TFA at 1 ml/min. Since the product peaks maximum at t_R = 19.6 min. is very close to the end of the method, part of the column purging procedure after t_R = 20 min. with 95 % acetonitrile in 0.1 % aqueous TFA is shown for completion.

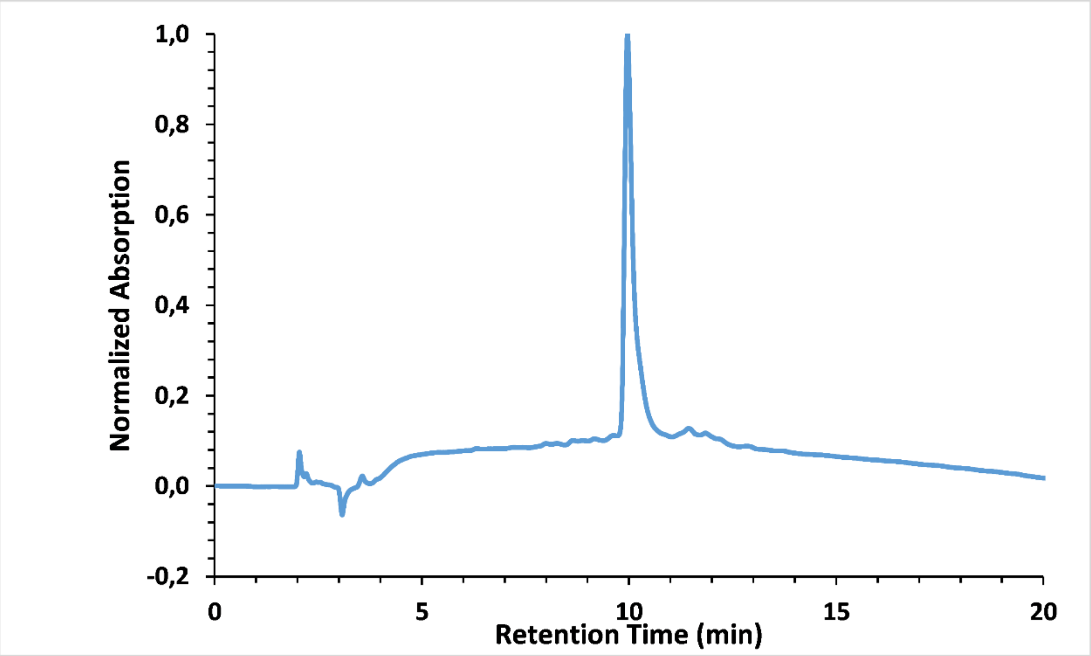

Figure S-28: RP-HPLC chromatogram at 214 nm of OctF3PrgAcid. Gradient of 20 % to 80 % acetonitrile over 20 minutes in 0.1 % aqueous TFA at 1 ml/min.

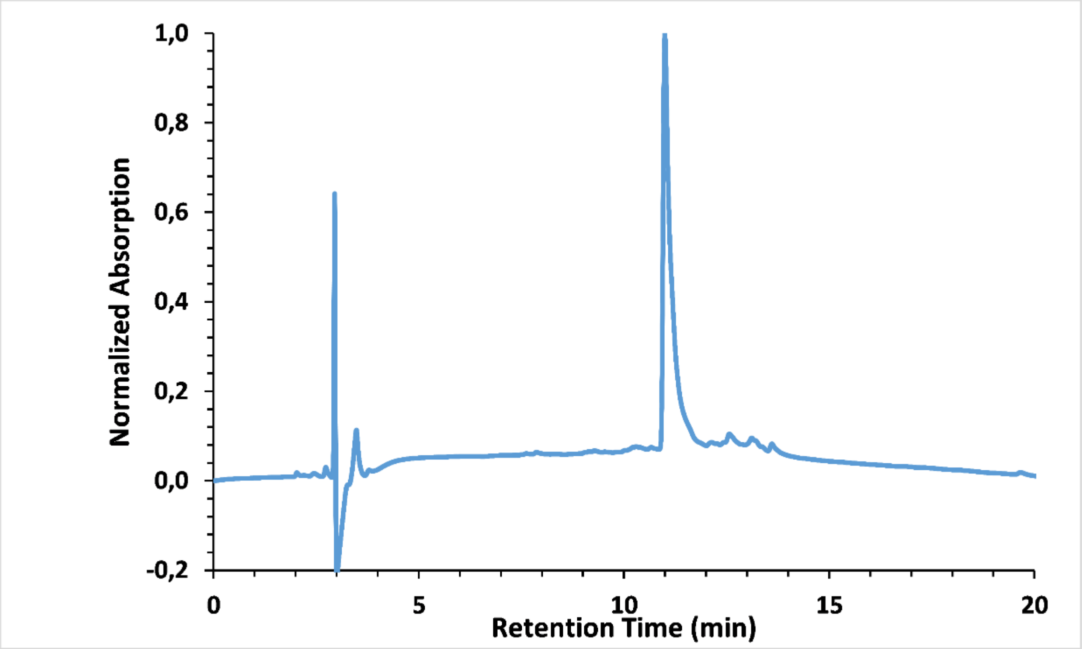

Figure S-29: RP-HPLC chromatogram at 214 nm of OctF1PrgAcid. Gradient of 20 % to 80 % acetonitrile over 20 minutes in 0.1 % aqueous TFA at 1 ml/min.

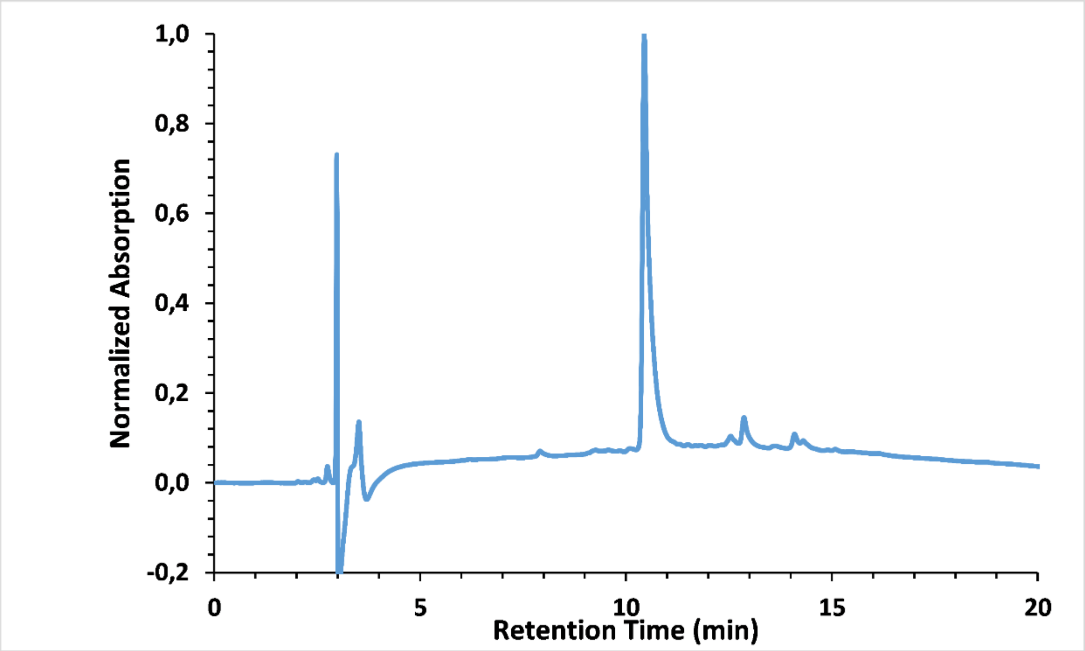

Figure S-30: RP-HPLC chromatogram at 214 nm of OctF3AllylAcid. Gradient of 20 % to 80 % acetonitrile over 20 minutes in 0.1 % aqueous TFA at 1 ml/min.

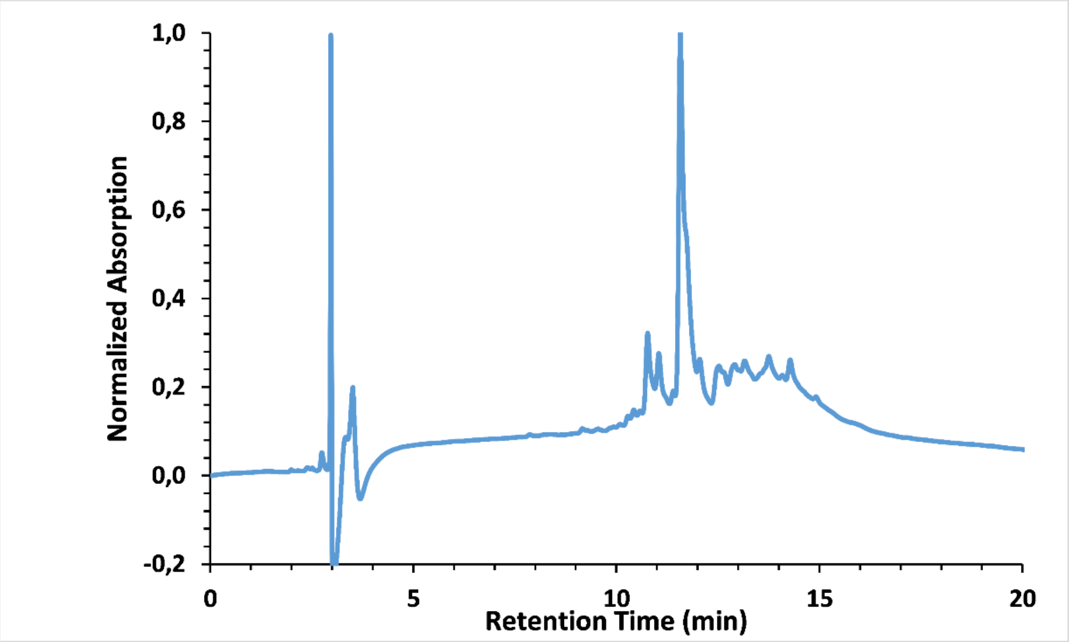

Figure S-31: RP-HPLC chromatogram at 214 nm of OctF1AllylAcid. Gradient of 20 % to 80 % acetonitrile over 20 minutes in 0.1 % aqueous TFA at 1 ml/min.

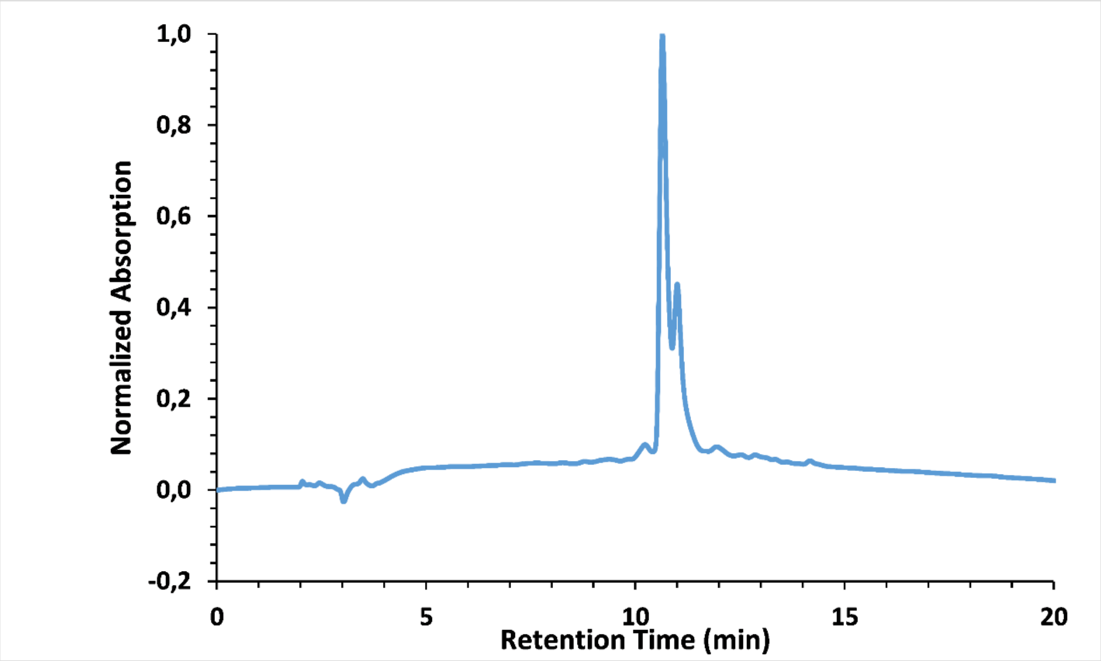

Figure S-32: RP-HPLC chromatogram at 214 nm of OctF1PrgAlcohol. Gradient of 20 % to 80 % acetonitrile over 20 minutes in 0.1 % aqueous TFA at 1 ml/min.

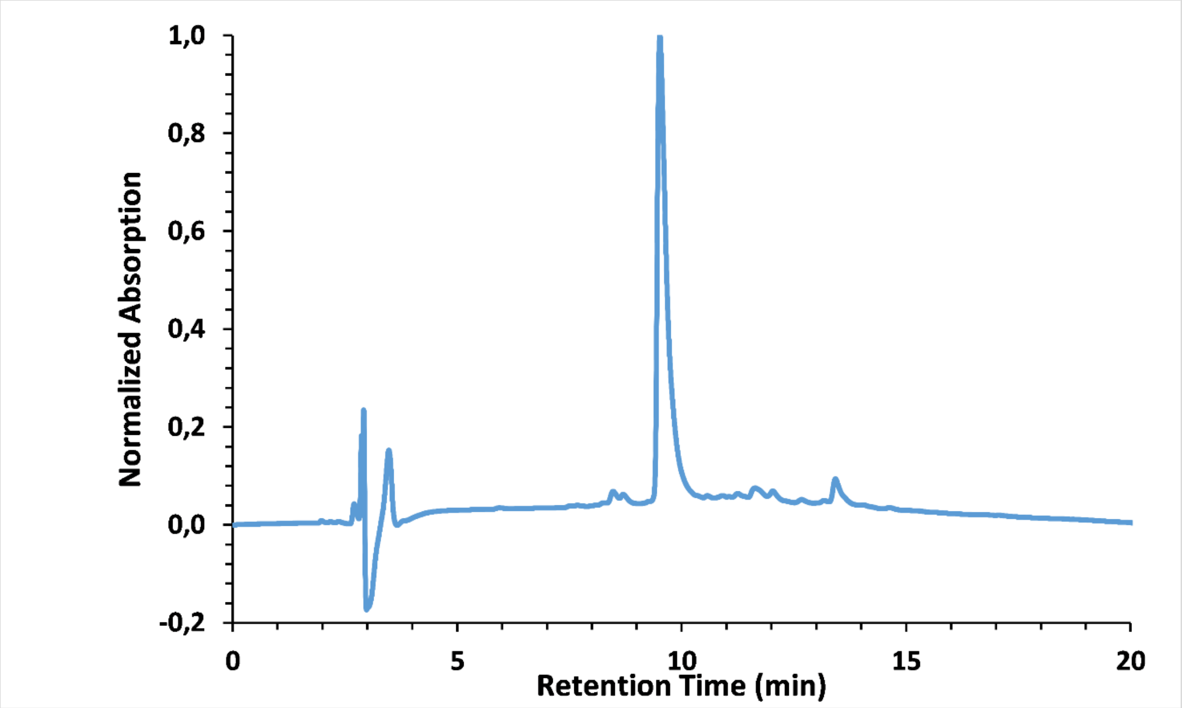

Figure S-33: RP-HPLC chromatogram at 214 nm of OctF3PrgAlcohol. Gradient of 20 % to 80 % acetonitrile over 20 minutes in 0.1 % aqueous TFA at 1 ml/min.

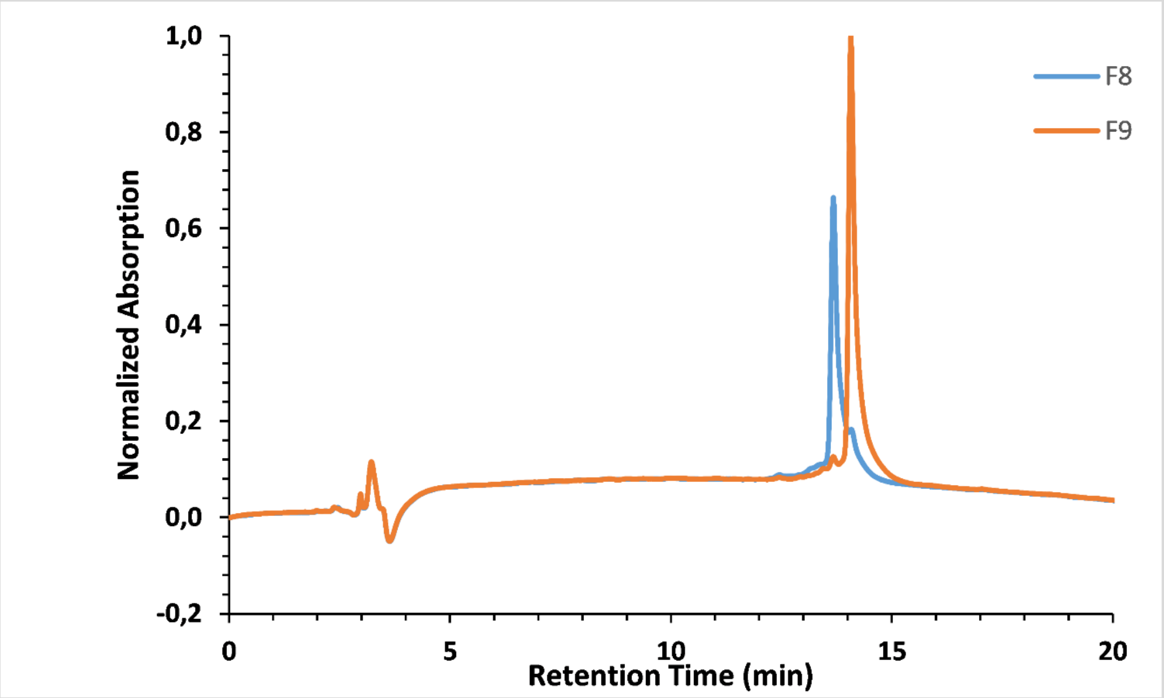

Figure S-34: RP-HPLC spectra at 214 nm of TAMRA-OctF3PrgAcid after preparative HPLC. Gradient of 20 % to 80 % acetonitrile over 20 minutes in 0.1 % aqueous TFA at 1 ml/min. Fractions F8 and F9 were united.

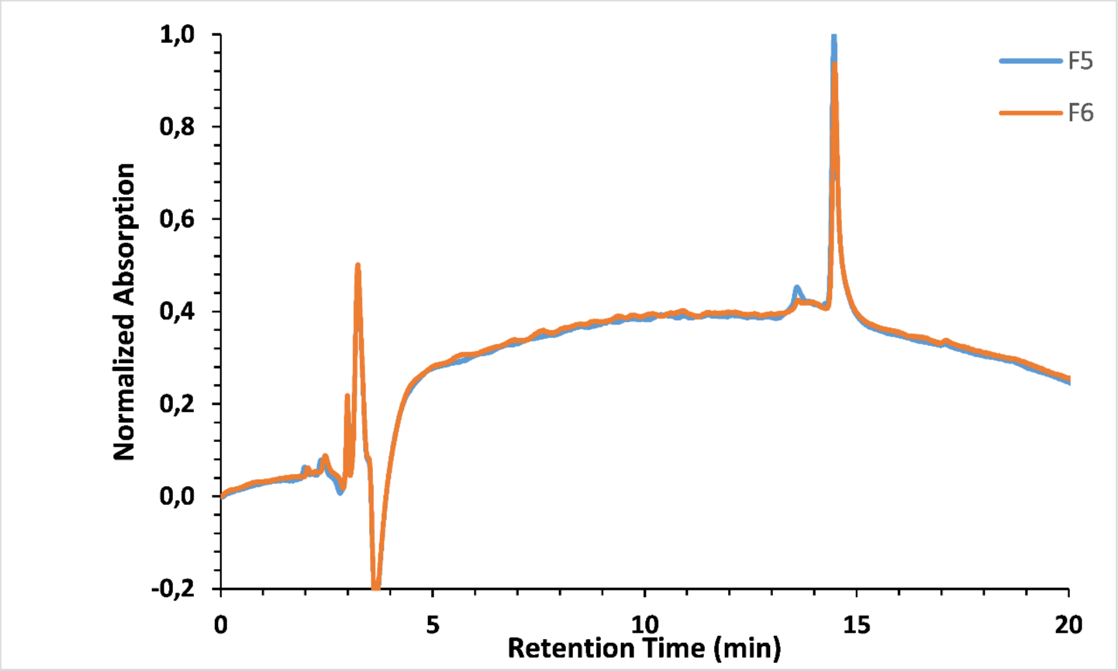

Figure S-35: RP-HPLC chromatogram at 214 nm of TAMRA-OctF1PrgAcid after preparative HPLC. Gradient of 20 % to 80 % acetonitrile over 20 minutes in 0.1 % aqueous TFA at 1 ml/min. Fractions F5 and F6 were united.

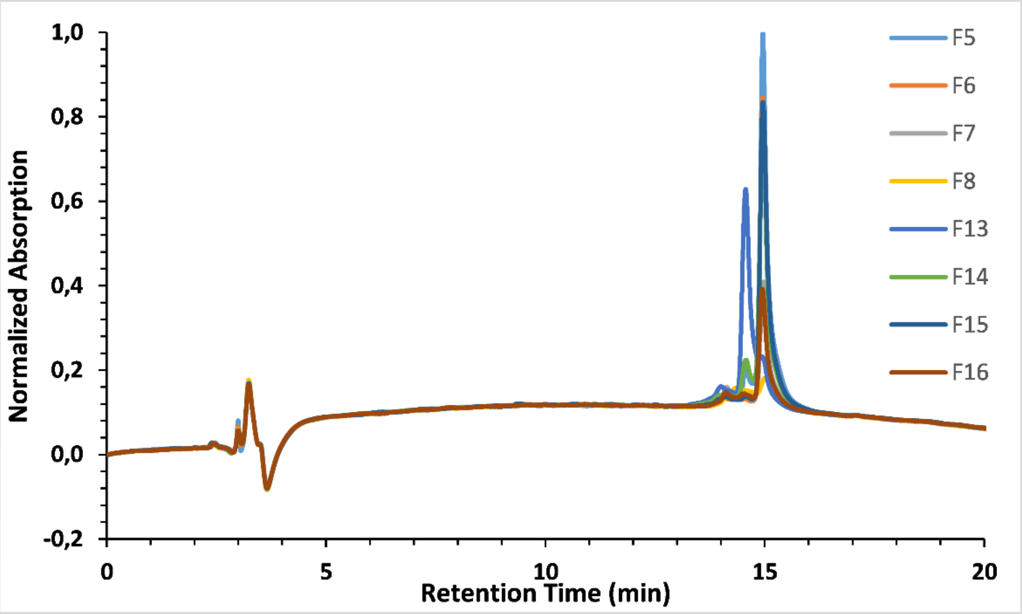

Figure S-36: RP-HPLC chromatogram at 214 nm of TAMRA-OctF3AllAcid after preparative HPLC. Gradient of 20 % to 80 % acetonitrile over 20 minutes in 0.1 % aqueous TFA at 1 ml/min. Fractions F5 to F8 and F13 to F16 were united.

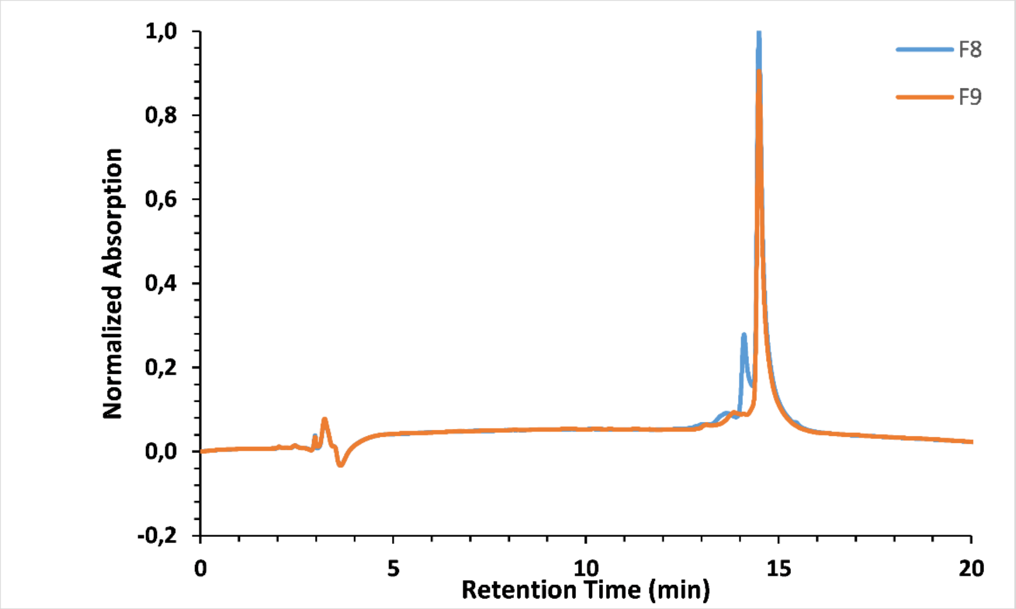

Figure S-37: RP-HPLC chromatogram at 214 nm of TAMRA-OctF1AllAcid after preparative HPLC. Gradient of 20 % to 80 % acetonitrile over 20 minutes in 0.1 % aqueous TFA at 1 ml/min. Fractions F8 and F9 were united.

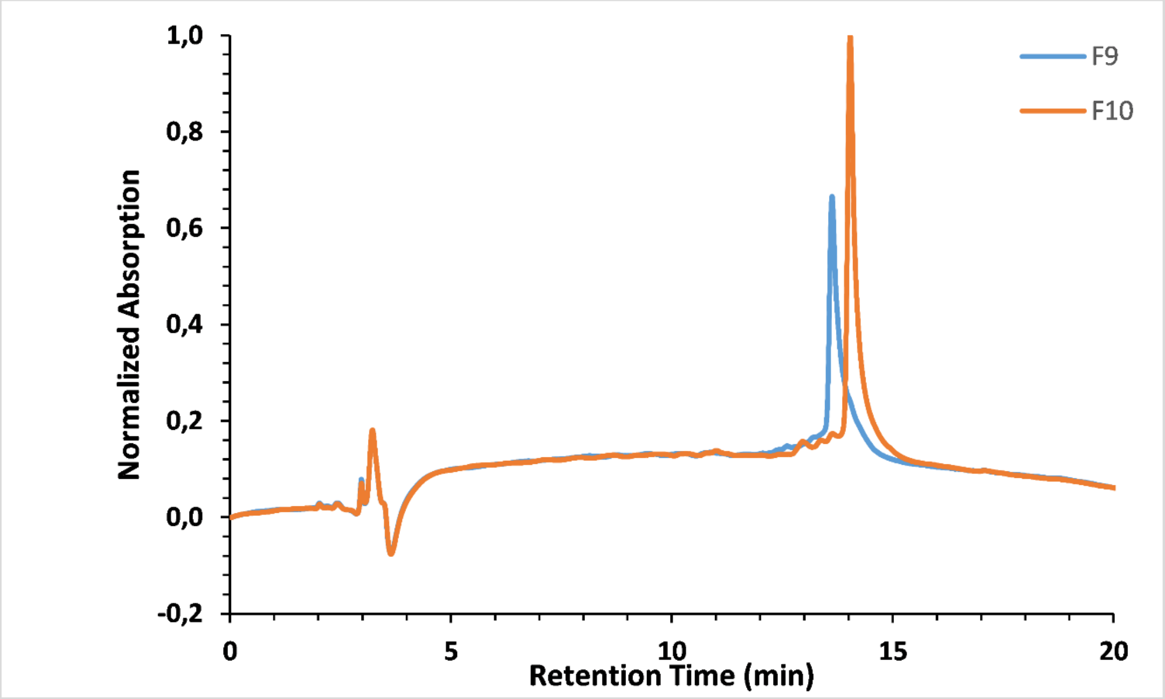

Figure S-38: RP-HPLC chromatogram at 214 nm of TAMRA-OctF3PrgAlcohol after preparative HPLC. Gradient of 20 % to 80 % acetonitrile over 20 minutes in 0.1 % aqueous TFA at 1 ml/min. Fractions F9 and F10 were united.

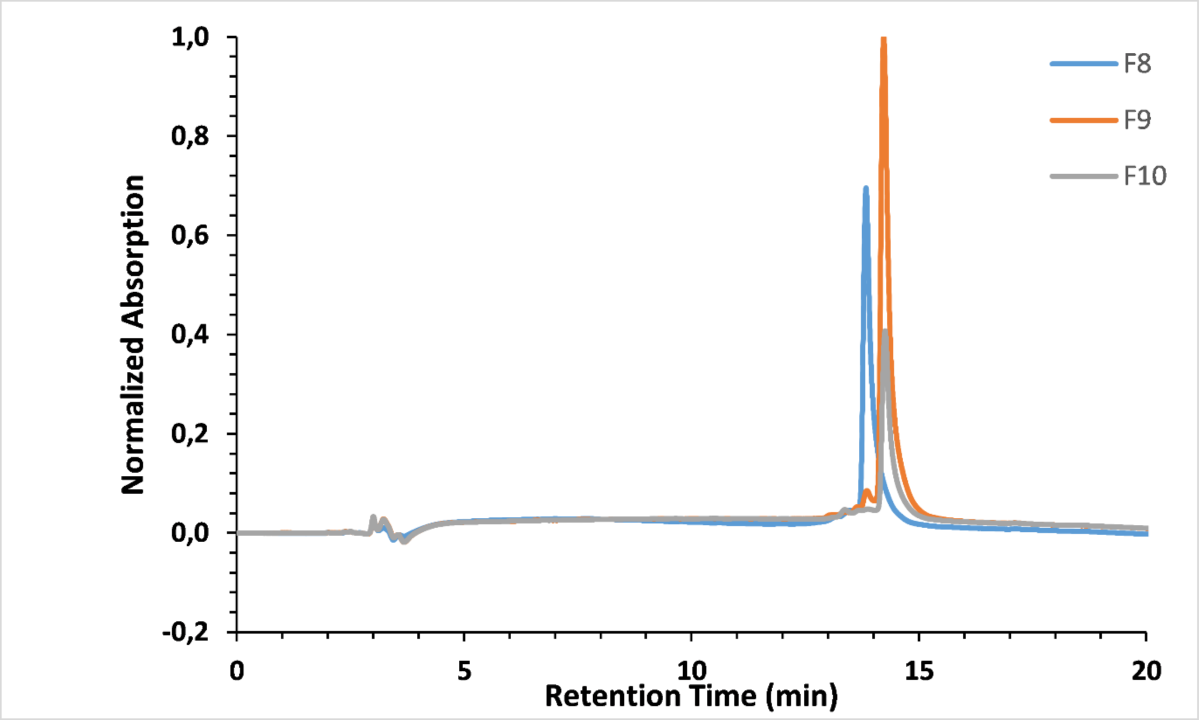

Figure S-39: RP-HPLC spectra at 214 nm of TAMRA-OctF1PrgAlcohol after preparative HPLC. Gradient of 20 % to 80 % acetonitrile over 20 minutes in 0.1 % aqueous TFA at 1 ml/min. Fractions F8 to F10 were united.

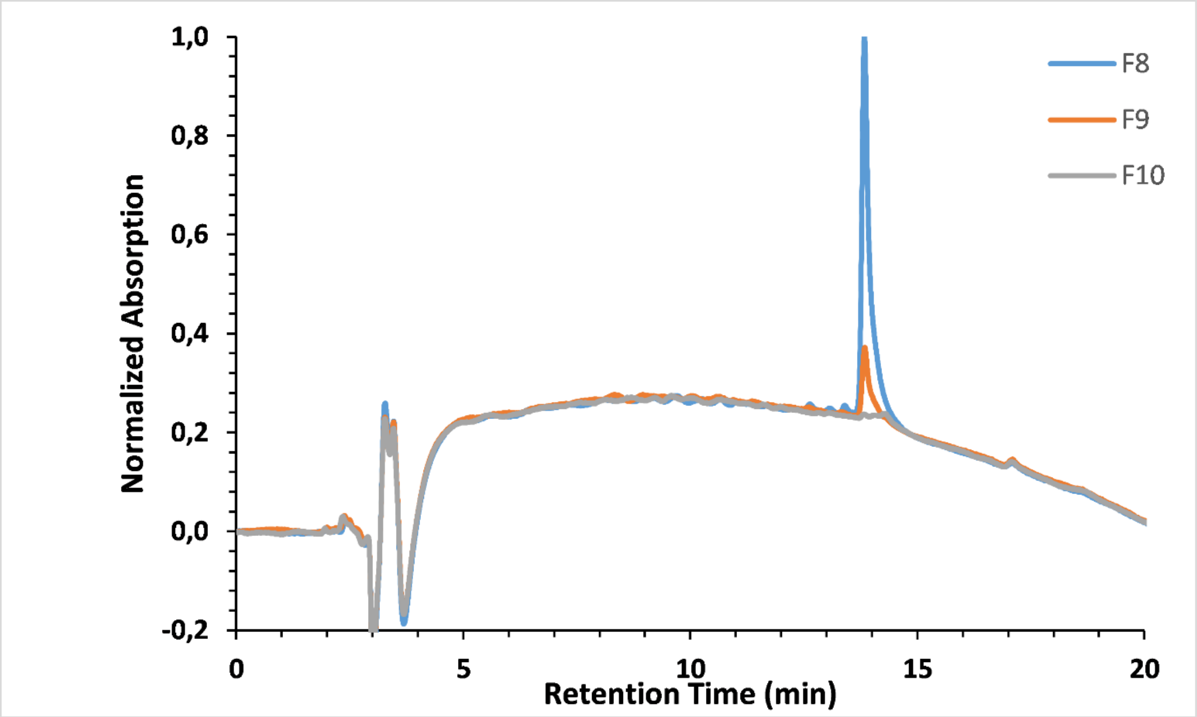

Figure S-40: RP-HPLC chromatogram at 214 nm of TAMRA-Octreotide after preparative HPLC. Gradient of 20 % to 80 % acetonitrile over 20 minutes in 0.1 % aqueous TFA at 1 ml/min. Fractions F8 to F10 were united.

References

1. Rodriguez, M., Llinares, M., Doulut, S., Heitz, A. & Martinez, J. A facile synthesis of chiral N-protected β-amino alcohols. *Tetrahedron Lett.* **32,** 923–926; 10.1016/S0040-4039(00)92121-X (1991).

2. Zhao, Z. G., Im, J. S., Lam, K. S. & Lake, D. F. Site-specific modification of a single-chain antibody using a novel glyoxylyl-based labeling reagent. *Bioconjug. Chem.* **10,** 424–430; 10.1021/bc980120k (1999).

3. Kvach, M. V. *et al.* Practical synthesis of isomerically pure 5- and 6-carboxytetramethylrhodamines, useful dyes for DNA probes. *Bioconjug. Chem.* **20,** 1673–1682; 10.1021/bc900037b (2009).

4. Merrifield, R. B. Solid Phase Peptide Synthesis. I. The Synthesis of a Tetrapeptide. *J. Am. Chem. Soc.* **85,** 2149–2154; 10.1021/ja00897a025 (1963).

5. García-Martín, F., Bayó-Puxan, N., Cruz, L. J., Bohling, J. C. & Albericio, F. Chlorotrityl Chloride (CTC) Resin as a Reusable Carboxyl Protecting Group. *QSAR Comb. Sci.* **26,** 1027–1035; 10.1002/qsar.200720015 (2007).

6. Wenschuh, H. *et al.* Stepwise Automated Solid Phase Synthesis of Naturally Occurring Peptaibols Using FMOC Amino Acid Fluorides. *J. Org. Chem.* **60,** 405–410; 10.1021/jo00107a020 (1995).

7. Sidorova, M. V. *et al.* Primenenie perekisi vodoroda dlia zamykaniia disul'fidnykh mostikov v peptidakh. *Bioorg. Khim.* **30,** 115–125; 10.1023/b:rubi.0000023093.05123.31 (2004).

8. Kiryutin, A. S. *et al.* A highly versatile automatized setup for quantitative measurements of PHIP enhancements. *J. Magn. Reson.* **285,** 26–36; 10.1016/j.jmr.2017.10.007 (2017).

9. Giernoth, R., Huebler, P. & Bargon, J. Intermediate Product-Catalyst Complexes in the Homogeneous Hydrogenation of Styrene Derivatives with Parahydrogen and Cationic RhI Catalysts. *Angew. Chem., Int. Ed.* **37,** 2473–2475; 10.1002/(SICI)1521-3773(19981002)37:18%3C2473::AID-ANIE2473%3E3.0.CO;2-J (1998).

10. Bowers, C. R. Sensitivity Enhancement Utilizing Parahydrogen. In *Encyclopedia of Magnetic Resonance,* edited by R. K. Harris (John Wiley & Sons, Ltd, Chichester, UK, 2007), Vol. 57, p. 696.

11. Giard, D. J. *et al.* In vitro cultivation of human tumors: establishment of cell lines derived from a series of solid tumors. *J. Natl. Cancer Inst.* **51,** 1417–1423; 10.1093/jnci/51.5.1417 (1973).

12. Graham, F. L., Smiley, J., Russell, W. C. & Nairn, R. Characteristics of a human cell line transformed by DNA from human adenovirus type 5. *J. Gen. Virol.* **36,** 59–74; 10.1099/0022-1317-36-1-59 (1977).
